# Patient Work and Their Contexts: Scoping Review

**DOI:** 10.2196/16656

**Published:** 2020-06-02

**Authors:** Kathleen Yin, Joshua Jung, Enrico Coiera, Liliana Laranjo, Ann Blandford, Adeel Khoja, Wan-Tien Tai, Daniel Psillakis Phillips, Annie Y S Lau

**Affiliations:** 1 Centre for Health Informatics Australian Institute of Health Innovation Macquarie University North Ryde Australia; 2 UCL Interaction Centre University College London London United Kingdom; 3 Department of Medicine Aga Khan University Karachi Pakistan

**Keywords:** self-care, burden of illness, self-management

## Abstract

**Background:**

Having patients self-manage their health conditions is a widely promoted concept, but many patients struggle to practice it effectively. Moreover, few studies have analyzed the nature of work required from patients and how such work fits into the context of their daily life.

**Objective:**

This study aimed to review the characteristics of patient work in adult patients. Patient work refers to tasks that health conditions impose on patients (eg, taking medications) within a system of contextual factors.

**Methods:**

A systematic scoping review was conducted using narrative synthesis. Data were extracted from PubMed, Excerpta Medica database (EMBASE), Cumulative Index to Nursing and Allied Health Literature (CINAHL), and PsycINFO, including studies from August 2013 to August 2018. The included studies focused on adult patients and assessed one or more of the following: (1) physical health–related tasks, (2) cognitive health–related tasks, or (3) contextual factors affecting these tasks. Tasks were categorized according to the themes that emerged: (1) if the task is always visible to others or can be cognitive, (2) if the task must be conducted collaboratively or can be conducted alone, and (3) if the task was done with the purpose of creating resources. Contextual factors were grouped according to the level at which they exert influence (micro, meso, or macro) and where they fit in the patient work system (the macroergonomic layer of physical, social, and organizational factors; the mesoergonomic layer of household and community; and the microergonomic triad of person-task-tools).

**Results:**

In total, 67 publications were included, with 58 original research articles and 9 review articles. A variety of patient work tasks were observed, ranging from physical and tangible tasks (such as taking medications and visiting health care professionals) to psychological and social tasks (such as creating coping strategies). Patient work was affected by a range of contextual factors on the micro, meso, or macro levels. Our results indicate that most patient work was done alone, in private, and often imposing cognitive burden with low amounts of support.

**Conclusions:**

This review sought to provide insight into the work burden of health management from a patient perspective and how patient context influences such work. For many patients, health-related work is ever present, invisible, and overwhelming. When researchers and clinicians design and implement patient-facing interventions, it is important to understand how the extra work impacts one’s internal state and coping strategy, how such work fits into daily routines, and if these changes could be maintained in the long term.

## Introduction

### Background

Chronic diseases are increasingly prevalent as the world’s population ages, requiring millions of patients to adjust their lifestyle and manage their health [1]. However, patient work and patient ergonomics, defined, respectively, as the combination of all health-related tasks and the contextual factors influencing the tasks [2], are given little attention. Existing studies have investigated the influences of individual barriers for health management, such as time requirements [3], the burden of comorbidity [4,5], or complexities in specific diseases [6-8]. Few studies have analyzed the character of patient work and how such work fits into the context of an individual’s life.

Patients conduct a variety of cognitive, visible, and collaborative work to accommodate health conditions and treatments, with such tasks changing throughout the *illness journey* as treatments are introduced or removed [9-13]. Using a work ergonomic system, Holden et al [2,14] posited that *patient work* is affected by a *patient work system,* which incorporates contextual factors affecting the performance of work [15-20]. A poor integration of patient work into the existing context and routine can generate excess stress, potentially contributing to noncompliance and suboptimal health outcomes [21,22]. The patient work system [2] groups all contextual factors into 3 levels: a microergonomic level (further separated into people, task, and tools), a mesoergonomic level including household and community, and a macroergonomic level (physical, social, and organizational), separating the influences that are inherent within the task or the patient from those that came from daily life.

Although health-related tasks are included in the umbrella terms of *self-management* (defined as actions and processes that people with a health problem intentionally perform to manage health in partnership with health care professionals [23]) and *self-care* (a more loose definition encompassing all things people do to manage and improve their health [24]), such tasks have not been reviewed to reflect how they relate to different aspects of the patient’s life. Similarly, there have been few attempts to assess how patient work is influenced by different contextual factors. In particular, although these tasks and factors have been explored in specific conditions [2,25], a review across all health conditions remains lacking. Therefore, a scoping review was chosen to rapidly gain an understanding of this nascent field, following guidance from Arksey and O’Malley [26], who noted that the method was appropriate “especially where an area is complex or has not been reviewed comprehensively before.”

### Objectives

In this scoping review, we review the existing literature on patient work, examining what people do in self-management and why they do or do not undertake certain tasks [2]. We also examine the different levels of patient work contexts, where digital interventions could play a supporting role. Specifically, digital technologies can offer health advice based on the immediate tasks and contexts around the patient [27], making digital apps a viable solution to supporting patient work through one’s life.

## Methods

### Search Strategy

The search was conducted using a modified participants, interventions, comparisons, and outcome strategy, which stated that the research question for a review must include the population, intervention, comparison, and outcome. Our research question was “What are the characteristics of patient work in adult patients?,” with the population being adult patients, intervention being the presence of a health condition, comparison being daily life before diagnosis, and the outcome being the characteristics of patient work. A search was conducted on August 23, 2018, in PubMed, Excerpta Medica database (EMBASE), Cumulative Index to Nursing and Allied Health Literature (CINAHL), and PsycINFO, including all articles published from August 2013 onward. The search terms were designed to capture publications that depicted work conducted by patients to maintain their health and how such work was limited or facilitated by contextual factors. Only articles published in English were included in the search. Multimedia Appendix 1 provides the complete search strategy.

### Inclusion and Exclusion Criteria

Articles were eligible if they focused on adult patients, included a qualitative component, and focused on assessing the impact of (1) physical tasks that patients undertake to manage their health, (2) cognitive tasks associated with managing health, *or* (3) contextual factors facilitating or restricting the physical or cognitive tasks.

Articles were excluded if they only focused on nonpatients (eg, caregivers); if they only presented biological or physiological data; if they only addressed health-related contextual factors that do not affect the work patients do (eg, computing systems in a hospital); were opinion articles or protocol papers; *or* if they focused on the design or evaluation of a measuring instrument, tool, or intervention.

### Study Screening and Data Extraction

Multimedia Appendix 1 provides details on abstract and full-text screening as well as data extraction from full-text articles. Each abstract and full-text was screened independently by 2 researchers, and each full-text article was also screened independently by 2 researchers. The interrater score for abstract screening was 0.39 (fair agreement) [28] and for full-paper screening was 0.30 (fair agreement) [28]. Disagreements were resolved by having a third independent reviewer review the conflicting article and make a final decision. Data extraction was conducted by 3 researchers who met regularly to address concerns and to ensure that data extraction was conducted consistently.

### Analysis Framework

We conducted a narrative synthesis on the patient work tasks and extracted contextual factors. The patient work tasks were assessed using the patient work model initially proposed by Corbin and Strauss [29,30], which indicates patient work as *illness work* that is influenced by *everyday life work* and *biographical work*. The work results in 3 types of tasks for self-management: medical management (eg, planning doctor appointments), emotional management (eg, dealing with anxiety and fear), and role management (eg, balancing one’s role in the family with one’s illness). We consulted a modified version of Corbin and Strauss’s model by Dack et al [31], which encompassed the 3 types of tasks for self-management. For contexts, we used the patient work system proposed by Holden et al [2,14]. The patient work system encompasses a microergonomic aspect, which describes how contextual factors from the patient, task, and tools involved [14,32] affect the work done; a mesoergonomic layer of household and community; and a macroergonomic aspect where physical, social, and organizational influences [2,14] are outlined.

Work tasks were identified from the included articles, were clustered based on common themes that emerged, and were consulted against Dack et al’s model [31]. Contextual factors were grouped according to where they fit within the dimensions outlined in the patient work framework (patient, tasks, tools, physical, social, and organizational) [2,14,32] and the level at which they exert influence (micro, meso, or macro) [33,34]. We identified microlevel contextual factors as aspects that only affected the patient’s body or were psychological factors. Mesolevel contextual factors were the influences of people socially close to the patient or influences exerted by the immediate physical surroundings or social circumstances (eg, finances) of the patient [35]. We identified macrolevel contextual factors as aspects that arose from the society, culture, or geopolitical entity in which the patient lived. The micro-meso-macro framework has been used extensively in health care, ranging from describing the quality of life assessments at different levels of decision making [36], patient-reported outcome measures in hospital palliative care [34], enablers and barriers affecting nursing practices [37], policy implementation of health leadership [38], to health care priority setting across different countries [39].

The articles were initially read by KY, JJ, and DP to extract passages that described patient work tasks or contextual influences on patient work. The 3 researchers then compiled our findings to reach a consensus and held frequent meetings over 1 month to sort all identified patient work tasks and contextual influences into themes. Reviewers decided by consensus to categorize the work tasks and the contextual factors based on the commonalities observed.

## Results

### Screening Process

**Figure 1:**
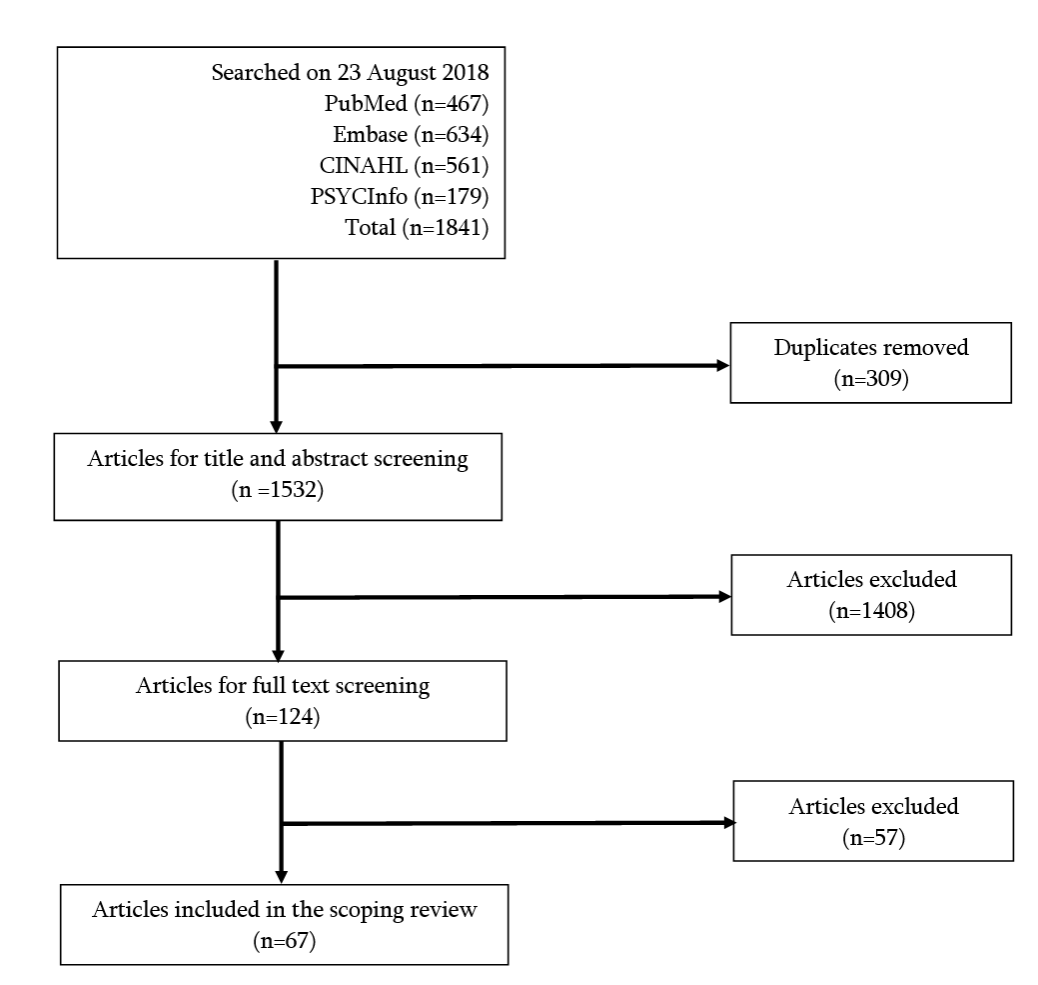
Number of articles included at each stage of the screening process.

Figure 1 outlines the screening process. Multimedia Appendix 2 gives more details about the screening process.

### Characteristics of Included Articles

Among the 67 articles included in the scoping review, 58 were original research and 9 were reviews. Semistructured interviews were the most common research strategy employed, and the articles studied 37 different health conditions. Multimedia Appendices 3 and 4 provide more information on the type and characteristics of the included articles.

### Patient Work Tasks Conducted by Participants

Patient work occurs in 2 forms: visible and cognitive work. Visible work is performed within a physical space (such as driving to visit doctors). Such tangible tasks could be observed by other people and are easy for others to intervene. Cognitive work, on the other hand, is completely unseen by others (eg, mentally counting calories throughout the day). Such tasks would not be revealed unless the patient discussed the information directly, and the task could remain hidden even from family and close friends.

Patient work tasks can also be conducted collaboratively or alone. Some tasks must be conducted in collaboration with others (eg, visiting health professionals), whereas cognitive tasks are always conducted alone (eg, developing mental coping strategies). Most patient work tasks, however, existed between these 2 extremes and could be conducted collaboratively or alone, depending on the contextual influences.

All patient work tasks also consumed resources such as time [40,41], physical energy [42], or social support. However, although many patient work tasks consumed resources to maintain one’s state of health, some tasks had the precise purpose of creating more resources, such as learning about one’s health condition or attending patient support group meetings. The prevalence of resources available to a patient serves as a buffer to mitigate the effect of sudden changes (eg, having enough funds to undergo surgery), and tasks that increase resources can ultimately increase the patient’s capacity to cope [43].

In total, 6 different categories of patient work tasks emerged along the 3 aforementioned axes and are listed in Textbox 1*.* The categories were distinctive based on whether (1) the task was always visible or not, (2) the task must be conducted collaboratively or not, and (3) the purpose of the task was to create resources or not.

Tasks were further divided into 21 specific types of tasks according to the reviewers’ consensus. Multimedia Appendix 5 provides a list of tasks identified in each included article, and Multimedia Appendix 6 provides a thorough description of each task type together with examples.

**Figure 2:**
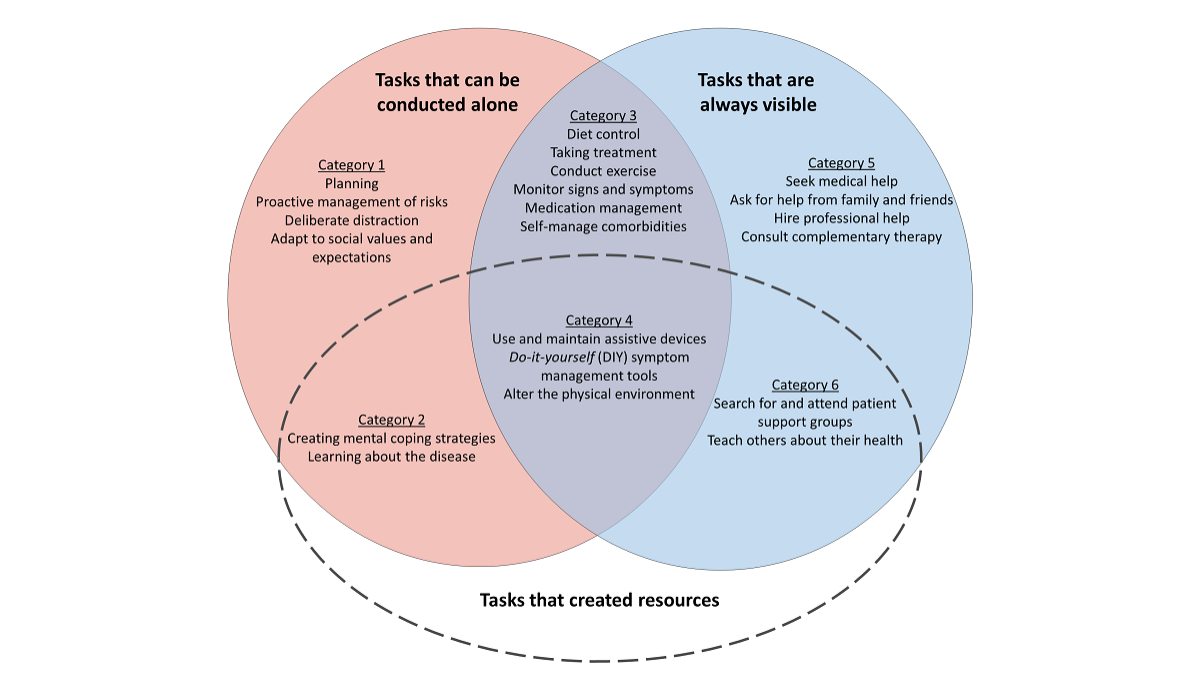
Patient work tasks were categorized according to their alignment along the 3 axes of collaboration, visibility, and creating resources.

Figure 2 demonstrates how the tasks fit into the 6 identified categories and the relationship of the categories with each other.

**Classification of patient work tasks.**

Category and description of the patient work tasks

- Category 1: Not always visible, can be conducted alone, and consumed resources
- Category 2: Not always visible, can be conducted alone, and purposefully created resources
- Category 3: Visible, can be conducted alone, and consumed resources
- Category 4: Visible, can be conducted alone, and purposefully created resources
- Category 5: Visible, must be conducted collaboratively, and consumed resources
- Category 6: Visible, must be conducted collaboratively, and purposefully created resources

#### Category 1: Tasks That Are Not Always Visible, Can Be Conducted Alone, and Consumed Resources

Category 1 tasks made up a large portion of the instances of patient work tasks identified in the included studies, with the most prevalent tasks being *planning* [18,20,40,44-57] and *proactive management of risks* [20,40-42,45-47,49,50,52,57-78]. Tasks in category 1 are characterized by their pervasiveness and volume. Patients are constantly thinking about their prospective health-related plans and mitigating health risks, even when they are not currently experiencing symptoms or remaining in a health care setting. Significantly, health-related work in this category includes tasks that do not necessarily improve health outcomes (such as *adapt to social values and expectations* [19,20,48,55,57,58,61,64,70,79-82]). Despite the volume of work in this category, patients do not often discuss such tasks with others, making such work unacknowledged and sometimes taken for granted by the patient and their families, receiving very little organizational or psychological support.

#### Category 2: Tasks That Are Not Always Visible, Can Be Conducted Alone, and Purposefully Created Resources

Work tasks included in category 2 are not so much about managing one’s symptoms, but work that enables patients to manage their symptoms better in the future. *Create mental coping strategies* [14,18,20,40,43,45,46,48-51,61,75-80,83-89] included overcoming emotional barriers [18], drawing upon spiritual beliefs [78], eventually coming to terms with a *new body* and a *new normal* [14,20,87,89] that enables patients to proactively engage with treatment. Patients also looked for relevant knowledge, either from written literature or by asking other people, in work tasks under *learning about the disease* [16,20,42,48,57,59,60,64,67,73,74,78,80,81,84,90]. Intellectual endeavors to understand the implications of symptoms [52] are also included here.

#### Category 3: Tasks That Are Visible, Can Be Conducted Alone, and Consumed Resources

Category 3 tasks included self-management tasks such as *diet control* [14,42,48,49,52,53,57,59,63-68,71,75,77-79,82,85,87, 90,91], *conduct exercise* [19,20,25,40-42,45,49,56,59,63,66, 67,73,77,79,82,87,90,91], *taking treatment* [2,18,42,47,49,57, 63,64,66-69,71,73-75,78,82,84,85,90-94], *monitor signs and symptoms* [2,14,41,42,44,45,57,65,67-69,71,85,87,90,92,95], *medication management* [40,43,44,51,52,57,59,71,75,84,90, 95,96], and *self-manage comorbidities* [14,41,44,59,75, 79,82,97]. The effective carrying out of tasks in this category is underpinned by category 1 and 2 tasks, benefiting from good planning and attitude changes. Although patients recognize this category to be a significant drain on their resources (whether in terms of time [40,41], physical capacity [42], or appropriate knowledge [19,47,71]), they are aware of the importance of such tasks and noted that support for such tasks is already in place from public health initiatives and health professionals.

#### Category 4: Tasks That Are Visible, Can Be Conducted Alone, and Purposefully Created Resources

Category 4 was the only category that explicitly described how patients changed their physical environment and tools. Tasks in this category (eg, *use and maintain assistive devices* [16,20,42,50,56,58,59,62,67,69,72,74,89,90,95,97,98], *do-it-yourself symptom management tools* [18,19,47,59,62, 66,74,77,95], and *alter the physical environment* [46,62,93]) had an immediate physical return in the form of better tools or a more comfortable physical environment, with the tools ranging from day-to-day items (eg, shoe insoles [97]) to specialized equipment (such as home oxygen tanks [59]). Patients are very aware of how this category is an investment to improve the quality of life as well as what kind of support (mainly financial) is available from health organizations.

#### Category 5: Tasks That Are Visible, Must Be Conducted Collaboratively, and Consumed Resources

This category included tasks where patients sought help from other people, including *seek medical help* [2,14,18,40,41,44,49-52,58,65,75,79,80,83,89,93,96,97], *ask for help from family and friends* [18,40,46,47,50,51,57,60, 63,67,69,73,77,93,95,98], *hire professional help* [20,43,52,69,95,98], and *consult complementary therapy* [58,59,64,66,84]. This category also included unplanned interactions, such as visits to the emergency department [41,89]. The patient reported only initiating these tasks when issues have escalated beyond their individual control [41,52,89] and seeking help became a necessity. Being the most visible tasks observable by health professionals, health care systems have traditionally paid close attention to these interactions.

#### Category 6: Tasks That Are Visible, Must Be Conducted Collaboratively, and Purposefully Created Resources

The last category, category 6, included tasks that contributed specifically to building new social resources for the patient, *searching for and attending patient support groups* [57,59,67,73,99], and *teaching others about their health* [2,51,57,71,89]. Patients actively create new social circles and recruit other people into their lives, whether by joining patient support groups or educating family and friends about their health [2,51,57,71,89]. In these interactions, the patient acts as either a peer or an expert, instead of the party receiving help. They proactively share their own health information and self-care strategies with others and act to support other patients along the *illness journey*.

### Contextual Factors Influencing Patient Work Tasks

We identified 17 types of contextual factors that influence patient work tasks. Multimedia Appendix 5 provides a list of contextual factors identified in each included article, and Multimedia Appendix 7 provides a thorough description of each contextual factor with examples. The contextual factors were grouped into how they fit within the patient work system (either in the macroergonomic triad of physical, social, and organizational factors or within the microergonomic triad of person-task-tools) [2,14,32]. As the patient work system has 3 layers, with households and communities comprising the mesoergonomic layer between the macro and microergonomic contexts, we also grouped contextual factors based on the level (macro, meso, or micro) at which they affected patient work tasks [33,34]. Although Holden et al [100] have identified *mesoergonomics* as the study of the relationship between variables in different layers of an ergonomics system, the *meso* contextual factors we identified here correspond to factors that sit between the micro- and macroergonomics levels.

#### Microlevel Contextual Factors

**Figure 3:**
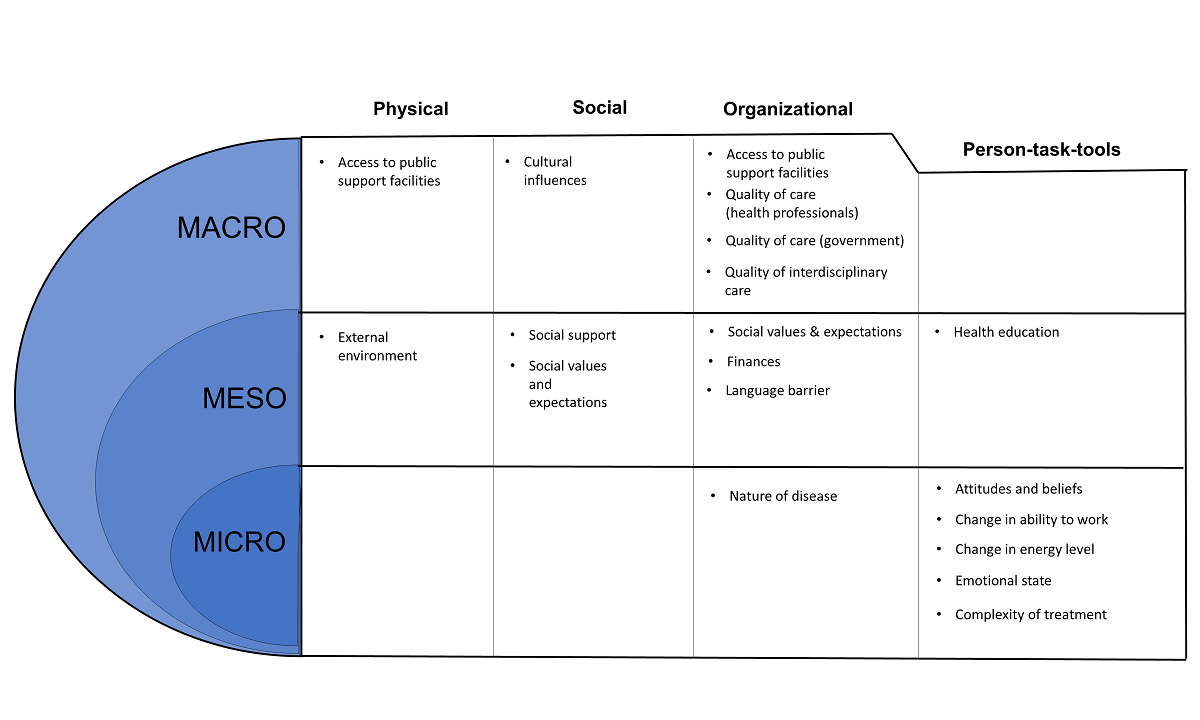
Contextual factors that influence patient work, separated on the basis of their allocation in the patient work system and whether they act on a macro, meso, or micro level.

We identified microlevel contextual factors as aspects that only affected health management by influencing a patient’s body or were psychological factors that arose from the patient’s mindset. Such factors significantly influence patients on a personal level and are included in the microergonomic triad of person-task-tools within the patient work system by Holden et al [2], as indicated in Figure 3. Microlevel factors in the review were mostly psychological factors, not always noticeable to family members or health professionals [2,45,61,70]. Such factors echo the type of patient work tasks that were not always visible, indicating a concrete need for resources that assist with psychological coping and adopting self-care into one’s routine.

#### Mesolevel Contextual Factors

Mesolevel contextual factors are aspects that affect self-management because of the influences of people socially close to the patient or influences exerted by the immediate surroundings or circumstances of the patient. These factors equally affected the physical, social, and organizational macroergonomic domains of the patient work system [2]. Contextual factors identified at this level, such as language barriers and social support, are widely recognized by health care systems, and supportive measures are often already in place [43,55,63,86]. Moreover, efforts to locally improve these contextual factors can significantly improve the patient’s health management, making such contextual factors an existing focus for intervention from health professionals.

#### Macrolevel Contextual Factors

Macrolevel contextual factors arise from the society, culture, or geopolitical entity in which the patient lived. These factors affected the organizational aspect of the patient work system [57,58,69,82]. Changing such contextual factors usually requires significant political or population health action, and individual patients and health care professionals often struggle to influence such factors on their own [47,57,63,69,82].

## Discussion

### Principal Findings

**Figure 4:**
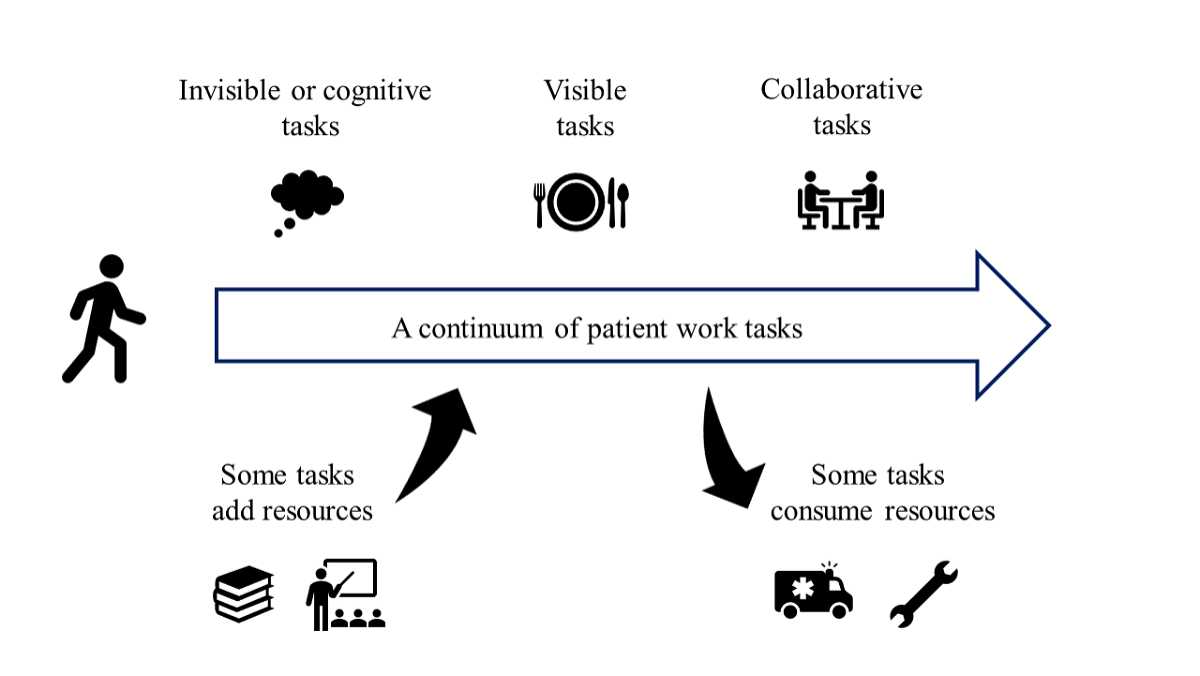
A continuum of patient work tasks that the patient moves through in their illness journey.

The purpose of this scoping review was to analyze the existing literature on the tasks that health conditions imposed on patients and the contextual factors affecting these tasks [2]. Our results indicate a continuum of patient work tasks, through which the patient moves from tasks that are cognitive only to tasks that are always visible and tasks that are always conducted collaboratively, while also experiencing how some tasks generated resources for the future, whereas others mainly consumed resources, as seen in Figure 4.

Contextual factors were mapped out by analyzing the factors according to their micro-, meso-, or macrolevel of influence and where they fitted along the patient work system, whether within the macroergonomic factors of physical, social, and organizational factors or the microergonomic triad of person-tasks-tool [14]. Although all contextual factors fit into the patient work system, there seems to be a *mesoergonomic* layer not specified in the patient work system that acted as a bridge between the macroergonomic influences and the more personal and psychological microergonomic contextual factors.

### Limitations

Our review has some limitations. We only included articles published in English and did not have access to studies in other languages. The relationship between the different aspects of work tasks is propositional and has not been empirically tested. We also focused on articles that addressed work conducted by patients themselves, as opposed to work conducted by caregivers.

Our review also did not cover detailed physical ergonomic factors of the patient’s vicinity, such as the physical properties of the areas where patients placed their medications, as the included articles did not contain these details.

### Comparison With Existing Literature

Corbin and Strauss [30] separated self-management into 3 different kinds of activities: emotional management, medical management, and role management. Category 1 (not always visible, can be conducted alone, and consumed resources) and category 2 (not always visible, can be conducted alone, and purposefully created resources) relate to cognitive workflow [101] and fit into emotional management within the Corbin and Strauss’s model. Within the cognitive workflow, which consists of sensemaking [102,103], planning, monitoring, decision making [9], and coordinating, the person makes the cognitive preparations necessary before conducting an action. This was reflected in activities such as planning and adjusting routines to *make space* for health-related work. Other cognitive tasks, such as finding information on the web and developing mental strategies, contribute to helping the patient to intellectually understand and psychologically accept their health condition.

Patients consider these tasks a part of life and rarely mention such tasks to health professionals despite its prevalence, reflecting a glaring need for more systematic support of these *invisible* tasks. Although this scoping review defined *not visible work* as work that is conducted primarily cognitively and thus *invisible* in the literal sense, tasks in categories 1 (not always visible, can be conducted alone, and consumed resources) and category 2 (not always visible, can be conducted alone, and purposefully created resources) can also fit into the definition of *invisible work* as it is used in ergonomics [104]—work that is taken for granted, underacknowledged, and undervalued. These tasks are only recognized as a burden when patients find them overwhelming.

A large body of literature exists on supporting patients with psychological distress, including psychotherapy [105] for patients with advanced cancer, cognitive behavioral therapy, and animal therapy [106], yet everyday tasks such as planning and establishing routines are much more nebulous to support. Patients reported using a variety of basic planning tools, such as notebooks and calendars with appointments written in them, and it is possible that a digital extension of such tools, such as integrated digital diaries, could better assist in this space.

Tasks in categories 3 (visible, can be conducted alone, and consumed resources) and category 4 (visible, can be conducted alone, and purposefully created resources) represent health-related work acted out by individual patients, corresponding to medical management [30]. The dimension of lifestyle changes [107] is incorporated in these tasks as patients carry out exercise, modify their diet, and sort their medications into doses, work that the clinician traditionally ascribes to self-management. It is well recognized that patients need to have sufficient resources such as time [40,41], access to affordable facilities, or physical capacities to perform tasks satisfactorily [42]. Health professionals and health organizations have focused on assisting with these tasks for many years, and many patients are aware of the presence of such support programs. Category 4 (visible, can be conducted alone, and purposefully created resources) present as a slightly different category that initially consumes time and money but ultimately results in a large increase in capacity, such as the purchase of assistive devices, which can significantly improve the efficacy of self-care in the future.

Tasks in categories 5 (visible, must be conducted collaboratively, and consumed resources) and category 6 (visible, must be conducted collaboratively, and purposefully created resources) are tasks that are inherently collaborative, bringing self-management out of the individual’s personal lives and interacting with role and relationship management [30] regarding health professionals or family. These tasks also correspond to the self-care dimensions of communication [107,108] and obtaining help [109]. Some tasks in category 5 have the patient play a passive role (eg, visiting the emergency department), whereas activities in category 6 saw the patient interact with others as a peer or an expert (eg, attending patient support groups and teaching others about the disease and treatments). When a patient acts as an equal partner in a health-related relationship and feels the relationship to be a positive and empowering experience, the psychological and social benefits expand the patient’s capacity [43] and social resilience [15,110]. However, although the health system noted the benefits of such tasks and encouraged collaborative and equal decision making between the patient and the clinician, category 6 tasks were the least prevalent in our review, indicating that such tasks may still be taken up by only a small section of more informed and proactive patients.

#### Contextual Factors in the Patient Work Framework

The patient work system [2] separates the patient’s surroundings into macroergonomic and microergonomic categories, with a middle household and community levels. In the macroergonomics layer, physical contexts described influences of the physical world and social contexts described the influence of other people, whereas organizational contexts encompassed temporal organization (daily routines), societal organization (finances and family roles), and political organization (health system and legal issues). In the microergonomics layer, person described the characteristics of the people involved, task described the inherent challenges of the task, and tools described the method or tool used to perform the task.

This review further separated the contextual influences into 3 layers: macro, meso, and micro. At the macrolevel, the patient work system addressed many factors that affected patients on a governmental or cultural level. The mesolevel, which was not explicitly stated in the patient work system, appears to act as a bridge transporting macrolevel values and expectations down to the individual. For example, cultural values filtered down to social expectations, and the structure of the health care system emphasized the impact of financial problems. Although not addressed within the patient work system, the meso layer certainly exists and exerts its influence through the people and the environment closest to the patient.

On the microlevel, most of the identified contextual factors fit into the person-task-tools triad, and most of those factors were within *person*. Patients in our review reported a variety of emotional and mental influences that altered their actions, indicating their motivation and belief in their capacity was sometimes more important than the resources available to them. Patients experiencing different health conditions were affected by common emotional states, such as fear of medication side effects and dreading the future [67,69,71,75,80]. As an element of concern, patients in our scoping review rarely reported consulting mental health professionals about these fluctuating emotions, opting instead to *brave through* on their own [45,58,60,71,75,96]. Although there is literature indicating that different personality types respond differently to intervention styles [111-113], it is also probable that patients need to be aware of the available psychological help or be motivated to use such services to receive sufficient help through their psychological journey.

### Conclusions

This review aimed to provide insight into the design and implementation of self-management interventions by understanding where the health-related work burden lies for community-based patients and how context influences such work. In our scoping review, we found a high prevalence of patient work tasks conducted in the privacy of the patient’s own lives—often alone, cognitive or unacknowledged, and consuming resources. However, despite continuous efforts to improve community-based self-management, there are no definitive models of intervention addressing this need, and patients continue to struggle to incorporate self-management into their daily lives.

Innovative digital technologies, such as using digital devices to track and monitor one’s health, may play a role in supporting individuals with these invisible and solitary health tasks. Similarly, more personalized and flexible treatment might be achieved by sociotechnical interventions, which carefully consider how interactions with the health care system could affect different facets of an individual’s daily life.

Existing self-management literature, traditionally only focusing on the physical aspects of health-related work, has been enriched by unveiling the internal experiences of patients. As research increasingly considers health as an interplay between biology, psychology, and sociology, we are beginning to better assess the various layers of patient work and contexts that can influence the implementation of self-care.

## Appendix

Multimedia Appendix 1 Search terms and screening processes.

Multimedia Appendix 2 Screening results.

Multimedia Appendix 3 Characteristics (original research articles).

Multimedia Appendix 4 Characteristics (reviews).

Multimedia Appendix 5

Multimedia Appendix 6 Example of patient worktasks.

Multimedia Appendix 7 Examples of contextual factors.
