# Supplementary material for: Patient Work and Their Contexts: Scoping Review
Source: J Med Internet Res. 2020 Jun 2;22(6):e16656. doi: 10.2196/16656 (PMC7298639; doi:10.2196/16656)
Supplement: Multimedia Appendix 1 [file jmir_v22i6e16656_app1.docx]

**Search strategy:**

(“patient work” OR “Self-Management” [Mesh] OR "Self Care" [Mesh]) AND (“Ergonomics” [Mesh] OR “Activities of Daily Living”[Mesh] OR “Patient Capacity” OR “Patient Experience”)

Limited to:

- English language

- Published in the last 5 years

- Humans

- Abstract available

**Study Screening:**

All publications retrieved from the database search on 23 August 2018 were imported into EndNote (version X8, Clarivate Analytics). Duplicates were found within EndNote and removed.

The abstracts of the retrieved publications were screened by 8 independent, blind reviewers, with each abstract screened by 2 reviewers. If disagreements arose, a third reviewer was asked to make the final decision. Publications that passed abstract screening were downloaded as full texts. The Cohen’s kappa statistic for abstract screening was 0.39 (fair agreement).

The full text publications were screened by 7 independent, blind reviewers, with each full text reviewed by 2 reviewers. Similar to abstract screening, a third reviewer was asked to make a decision in the case of disagreements. Following full text screening, 67 publications were included in the scoping review. The Cohen’s kappa statistic for full text screening was 0.30 (fair agreement).

Data extraction

Descriptive data regarding characteristics of the included publications were extracted by 3 reviewers. Characteristics extracted from primary research articles included the health condition studied; study methodology; duration of study; sample size; age of participants; gender ratio; nationality or ethnicity of the participants; participants’ duration of disease; whether the participants were co-habiting with others; and participants’ employment status.

For reviews, characteristics extracted included the type of review; which databases were searched; the timeframe of the search; number of studies included; number of participants included in the individual studies; nationalities of the included studies; and the study methods employed by the included studies.

Patient work tasks and contextual factors

Information regarding patient work tasks and contextual factors were extracted. These features were extracted by 3 researchers, who each reviewed one third of the included publications and labelled all tasks and contextual factors they could identify. These tasks and contextual factors were then combined, and those that were similar to each other were merged where appropriate. The inclusion and wording of each task and contextual factor were discussed amongst the trio until consensus was reached.
