# Supplementary material for: Patient Work and Their Contexts: Scoping Review
Source: J Med Internet Res. 2020 Jun 2;22(6):e16656. doi: 10.2196/16656 (PMC7298639; doi:10.2196/16656)
Supplement: Multimedia Appendix 2 [file jmir_v22i6e16656_app2.docx]

**Screening results:**

The search strategy yielded 1,841 publications, with 467 publications from PubMed, 634 from Embase, 561 from CINAHL, and 179 from PSYCInfo. Initial screening in EndNote identified and removed 309 duplicates. We then screened 1,532 abstracts against the eligibility criteria and excluded 1,408 abstracts. The remaining 124 publications were downloaded as full texts and screened against the eligibility criteria, and 57 full text publications were considered ineligible. At the conclusion of the entire screening process, 67 publications were included in the scoping review. Among the 67 publications included, 58 were original research articles and 9 were reviews.
