# Supplementary material for: Patient Work and Their Contexts: Scoping Review
Source: J Med Internet Res. 2020 Jun 2;22(6):e16656. doi: 10.2196/16656 (PMC7298639; doi:10.2196/16656)
Supplement: Multimedia Appendix 3 [file jmir_v22i6e16656_app3.docx]

The original research articles included data from adult patients aged 18 to 92 years old, were conducted in 20 different countries, and involved 37 different health conditions.

Out of the 58 original research articles, most participants were residents in the USA (22%) and UK (19%), followed by Sweden (16%) and Australia (14%). Combined, articles originating from these 4 countries make up 71% of the original research articles included, presenting data from a predominantly North European population. The most frequent type of health condition studied in the included articles reflected disease prevalence in these countries, with the most common condition studied being cardiovascular diseases (16%), followed by endocrine diseases (14%) and respiratory diseases (12%). The most frequently studied single health condition was diabetes (Type 1 and 2 combined).

In terms of methodology, semi-structured interview was the most common research strategy employed (43%), followed by multiple qualitative methods (24%) and unspecified interviews (16%). Only 2 articles used surveys/questionnaires exclusively. The studies had predominantly small sample sizes due to their qualitative nature, with 27 articles (47%) having n=10~19, followed by 12 articles (20%) having n=20~29. The smallest sample size was 8 participants and the largest was 260, with the latter carried out via questionnaire.

Similar number of articles recruited more males (18 articles, 31%) compared to studies that recruited more females (22 articles, 38%). A further 5 articles contained an even split of male to female participants, while 1 article (2%) contained only male participants, 6 articles (10%) contained only females, and 6 articles (10%) did not mention participant gender ratios.

Many articles (71%) did not mention the participants’ duration of disease. From the articles that did report it, the duration of disease ranged from 3 months to 43 years. 4 articles (7%) had a mean disease duration of 0-9 years, 9 articles (15%) had 10-19 years, and 4 articles (7%) had 20-29 years.

The age of participants was not specified for 28 articles (48%). For articles that indicated the participants’ age, 8 articles (14%) had a mean age of 49 years or younger, 12 articles (20%) had a mean age of 50-59 years, 5 articles (9%) had 60-69 years, and 5 articles (9%) had a mean age of 70 years or greater, presenting a population that is predominantly middle-aged or older.

Participant employment was stated in 21 articles (37%), with 11 articles (19%) stating the majority of the participants were unemployed, 6 articles (10%) stated the majority of participants were employed, 2 articles (3%) had an even split between employed and unemployed participants, while 1 article (2%) stated all participants were employed and 1 article (2%) stated all participants were unemployed.

Co-habitation was not mentioned in 40 (69%) of the articles. 15 articles (26%) stated most of their participants are co-habiting with other family members, while the remaining 3 papers (5%) had the majority of the participants living alone.

| **Study author(s) / year published** | **Health condition(s)** | **Methodology / duration** | **Sample size / characteristics** | |
| --- | --- | --- | --- | --- |
| Apps L D et al. (2015) | Asthma | Methodology:   - Semi-structured interviews   Interview duration: N/A  Repeated interviews = N | N = 29  Age: Mean = 49.45 years  Gender: 13 males, 16 females  Nationality/ethnicity: UK | Disease duration: N/A  Cohabitation: N/A  Employment status: N/A |
| Arman M (2015) | Chronic pain  (fibromyalgia, fatigue, musculoskeletal pain) | Methodology:   - Interviews   Interview duration: 35-85 minutes  Repeated interviews = N | N = 10  Age: Mean = 49 years  Gender: All female  Nationality/ethnicity: Swedish | Disease duration: N/A  Cohabitation: N/A  Employment status: All employed |
| Au T S et al. (2014) | Chronic orofacial pain | Methodology:   - Semi-structured interviews   Interview duration: 60 minutes  Repeated interviews = N | N = 25  Age: 65-83 years  Gender: 4 males, 21 females  Nationality/ethnicity: Hong Kong | Disease duration: N/A  Cohabitation: N/A  Employment status: N/A |
| Blandford A et al. (2015) | Kidney failure | Methodology:   - In-home observation of dialysis - Interviews with patients (n = 19)   Interview duration: N/A  Repeated interviews = N | N = 19  Age: 24-77 years  Gender: N/A  Nationality/ethnicity: UK | Disease duration: N/A  Cohabitation: N/A  Employment status: N/A |
| Bowling C B et al. (2017) | Chronic Kidney Disease | Methodology:   - Focus groups - Semi-structured interviews   Interview duration: N/A  Repeated interviews = N | N = 30  Age: >70 years (Mean = 75)  Gender: 29 males, 1 female  Nationality/ethnicity: USA (Atlanta, Georgia) / 60% black | Disease duration: N/A  Cohabitation: N/A  Employment status: N/A |
| Browne C et al. (2015) | Multiple sclerosis and bladder dysfunction | Methodology:   - Semi-structured interviews   Interview duration: 90 minutes  Repeated interviews = N | N = 19  Age: 37-64 years (Mean = 53.42)  Gender: 8 males, 11 females  Nationality/ethnicity: Irish | Disease duration: Mean = 17.55 years  Cohabitation: N/A  Employment status: N/A |
| Bukhave E B et al. (2014) | Hand osteoarthritis | Methodology:   - 43 interviews (including semi-structured and additional photo-interviews)   Interview duration: N/A  Repeated interviews = N | N = 31  Age: 38-89 years  Gender: 5 males, 26 females  Nationality/ethnicity: Danish | Disease duration: N/A  Cohabitation: N/A  Employment status: N/A |
| Burnett K et al. (2018) | Idiopathic pulmonary fibrosis (IPF) | Methodology:   - Semi-structured interviews (telephone)   Interview duration: 28 minutes  Repeated interviews = N | N = 100  Age: 57-90 years  Gender: 61 males, 39 females  Nationality/ethnicity: Australian | Disease duration: N/A  Cohabitation: N/A  Employment status: N/A |
| Cameron M H et al. (2013) | Multiple sclerosis | Methodology:   - EDSS (Expanded disability status scale) - FPSS (Fall prevention strategy survey) - Questionnaire - Fall calendars | N = 58  Age: 18-50 years  Gender: N/A  Nationality/ethnicity: USA (Portland, Oregon) | Disease duration: N/A  Cohabitation: N/A  Employment status: N/A |
| Chen K et al. (2016) | Chronic Obstructive Pulmonary Disease (COPD) | Methodology:   - Semi-structured interviews   Interview duration: 30-70 minutes  Repeated interviews = N | N = 19  Age: Mean = 74.42 years  Gender: All male  Nationality/ethnicity: Taiwanese | Disease duration: Diagnosis of COPD for >1 year  Cohabitation: 17 cohabiting  Employment status: N/A |
| Cheung M M Y et al. (2018) | Asthma | Methodology:   - Survey - Lung function assessment - Drawing activity: Participants freely drew their experience of asthma with no time limit. - Semi-structured interview   Interview duration: N/A  Repeated interviews = N | N = 18  Age: 19-68 years (Mean = 33.2)  Gender: 3 males, 15 females  Nationality/ethnicity: Australian / Caucasian (n=8), Asian (n=7), Other (n=3) | Disease duration: 0-11 years (n=12), 12-18 years (n=3), 18+ years (n=3)  Cohabitation: N/A  Employment status: 6 employed (3 teachers, 2 healthcare industry, 1 art industry), 9 unemployed (7 students, 2 retired), 3 other |
| Close C et al. (2015) | Low back or pelvic pain | Methodology:   - Interview   Interview duration: N/A  Repeated interviews = N | N = 14  Age: Mean = 33 years  Gender: All female  Nationality/ethnicity: UK | Disease duration: N/A  Cohabitation: N/A  Employment status: N/A |
| Cobussen-Boekhorst H et al. (2016) | Chronic bladder disease, overactive bladder, other bladder problems with the need of catheterisation | Methodology:   - Semi-structured interviews   Interview duration: 45-69 minutes  Repeated interviews = Y, after one week, three months and twelve months | N = 11  Age: 33-77 years (Mean = 57)  Gender: 6 males, 5 females  Nationality/ethnicity: Dutch | Disease duration: N/A  Cohabitation: 10 cohabiting, 1 living alone  Employment status: 6 employed, 5 unemployed |
| Czuber-Dochan W et al. (2013) | Inflammatory bowel disease-fatigue | Methodology:   - Focus group interviews (4-12 participants per group)   Focus group duration: 90-120 minutes | N = 46  Age: 27-80 years (Mean=55)  Gender: 15 males, 31 females  Nationality/ethnicity: UK | Disease duration: 0.25 – 50 years (Mean=20.74)  Cohabitation: N/A  Employment status: N/A |
| Dahlviken R M et al. (2015) | Takotsubo cardiomyopathy (TTC) | Methodology:   - Semi-structured interviews   Interview duration: 16-63 minutes  Repeated interviews = N | N = 14  Age: Mean = 67 years  Gender: All female  Nationality/ethnicity: Norwegian | Disease duration: N/A  Cohabitation: 13 cohabiting  Employment status: 6 employed, 7 unemployed, 1 disabled |
| Dal Bello-Haas et al. (2014) | Dementia | Methodology:   - Questionnaire - Depression scale - Trail Making Test Part B - Stroop interference - Semantic fluency - Phonemic fluency task - Clock drawing test - Repeatable Battery for the Assessment of Neuropsychological Status - Mini-Mental Status Exam - Functional Assessment Questionnaire - Berg Balance Scale | N = 260  Age: 44-97 years (Mean = 74)  Gender: 100 males, 160 females  Nationality/ethnicity: Canadian | Disease duration: N/A  Cohabitation: N/A  Employment status: N/A |
| Dehghanzadeh S et al. (2017) | Heart failure and living with cardiac resynchronisation therapy (CRT) | Methodology:   - Semi-structured in-depth interviews   Interview duration: 30-60 minutes  Repeated interviews = N | N = 17  Age: 25-93 years (Mean = 58)  Gender: 9 males, 8 females  Nationality/ethnicity: Iranian | Disease duration: Mean time since implant = 2 years  Cohabitation: N/A  Employment status: 4 housewives, 3 disabled from work |
| Dial M et al. (2018) | Obesity | Methodology:   - Review of literature - Semi-structured interviews   Interview duration: 18 minutes (average)  Repeated interviews = N | N = 14  Age: Male (Mean) = 48 years, Female (Mean) = 52 years  Gender: 8 males, 6 females  Nationality/ethnicity: USA / Caucasian | Disease duration: N/A  Cohabitation: N/A  Employment status: N/A |
| Ferreira S L et al. (2013) | Sickle cell disease | Methodology:   - Semi-structured interviews   Interview duration: N/A  Repeated interviews = N | N = 12  Age: 29-39 years  Gender: N/A  Nationality/ethnicity: Brazilian (Bahia) / 91.6% respondents declared themselves black | Disease duration: N/A  Cohabitation: N/A  Employment: 7 employed, 5 unemployed |
| Flurey C A et al. (2013) | Rheumatoid arthritis (RA) | Methodology:   - Semi-structured interviews   Interview duration: 45-90 minutes  Repeated interviews = N | N = 15  Age: Mean = 51.1 years  Gender: 3 males, 12 females  Nationality/ethnicity: UK | Disease duration: > 2 years (Mean = 14.8)  Cohabitation: N/A  Employment status: N/A |
| Franklin Z C et al. (2016) | Chronic musculoskeletal pain | Methodology:   - Semi-structured interviews in a home or hospital setting   Interview duration: 45 minutes  Repeated interviews = N | N = 8  Age: Mean = 53.8 years  Gender: 2 males, 6 females  Nationality/ethnicity: UK | Disease duration: Mean = 17.3 years  Cohabitation: N/A  Employment status: All retired or unable to work due to pain |
| Fritz H (2014) | Diabetes | Methodology:   - Time-geographic diary - Semi-structured interviews - Participant-generated photography   Interview duration: N/A  Repeated interviews = N | N = 10  Age: 40-64 years  Gender: All female  Nationality/ethnicity: USA (Central North Carolina) / 70% identified as black | Disease duration: Mean = 13.4 years  Cohabitation: N/A  Employment status: 6 employed |
| Galacher K I et al. (2018) | Stroke patients | Methodology:   - Semi-structured interviews   Interview duration: 60 minutes  Repeated interviews = N | N = 29  Age: Mean = 68 years  Gender: 16 males, 13 females  Nationality/ethnicity: Scottish | Disease duration: N/A  Cohabitation: N/A  Employment status: N/A |
| Gerrish K et al. (2013) | Tuberculosis (Pulmonary and non-pulmonary TB) | Methodology:   - Interviews   Interview duration: 30-90 minutes at patients’ home, 30-60 minutes at patients’ workplace  Repeated interviews = N | N = 14  Age: N/A  Gender: 9 males, 5 females  Nationality/ethnicity: UK / of Somali heritage | Disease duration: N/A  Cohabitation: N/A  Employment status: N/A |
| Graffigna G et al. (2013) | Type-2 diabetes | Methodology:   - 2-week diaries - Narrative interviews   Interview duration: N/A  Repeated interviews = N | N = 29  Age: 41-71 years (Mean = 51)  Gender: 16 males, 13 females  Nationality/ethnicity: Italian | Disease duration: N/A  Cohabitation: 26 cohabiting (14 married empty nest, 12 married full nest), 3 living alone  Employment status: 10 employed, 19 unemployed (12 retired, 7 never employed) |
| Graham L J et al. (2013) | Diabetes (Type 1 or 2 not specified), vision-related conditions, cardiovascular conditions, musculoskeletal conditions, other health conditions  *Two participants with no mentioned chronic disease | Methodology:   - Interviews   Interview duration: N/A  Repeated interviews = N | N = 17  Age: 65-92 years (Mean = 74.2)  Gender: 8 males, 9 females  Nationality/ethnicity: Canadian | Disease duration: N/A  Cohabitation: 12 cohabiting, 5 living alone, 1 not mentioned  Employment status: N/A |
| Halls S et al. (2014) | Rheumatoid arthritis | Methodology:   - Semi-structured interviews   Interview duration: 30-80 minutes  Repeated interviews = N | N = 16  Age: 33-78 years (Mean = 57.3)  Gender: 5 males, 11 females  Nationality/ethnicity: UK | Disease duration: 1-27 years (mean = 11.5)  Cohabitation: N/A  Employment status: N/A |
| Hammarlund C S et al. (2017) | Polio | Methodology:   - Interviews   Interview duration: 60-90 minutes (average= 70 minutes)  Repeated interviews = N | N = 14  Age: 61-78 years (Mean = 70)  Gender: 7 males, 7 females  Nationality/ethnicity: Swedish | Disease duration: 9-43 years (Mean = 26)  Cohabitation: 11 cohabiting  Employment status: 4 employed |
| Holden R J et al.  (2016) | Chronic diseases | Multiple qualitative methods:   - Skype-based interviews - In-home interviews - Interviews (telephone) - Focus groups (conducted in clinic and hospital meeting rooms) - Surveys - In-clinic observations - In- clinic interviews - Medical record reviews   Interview/focus group duration: N/A  Repeated interviews = N | N = N/A  Age: >18 years  Gender: N/A  Nationality/ethnicity: USA | Disease duration: N/A  Cohabitation: N/A  Employment status: N/A |
| Holden R J et al. (2015) | Heart failure | Methodology:   - Semi-structured interviews - Observations - Surveys - Medical record review   Interview duration: N/A  Repeated interviews = Y | N = 30  Age: 65-86 years (Mean = 74)  Gender: 17 males, 13 females  Nationality/ethnicity: USA / 60% white non-Hispanic, 33% black and 7% mixed-race | Disease duration: Mean = 6.84 years  Cohabitation: N/A  Employment status: 1 employed, 29 unemployed (26 retired, 3 disabled) |
| Jacobsson L R et al. (2017) | Coeliac disease | Methodology:   - Semi-structured interviews   Interview duration: 50 minutes  Repeated interviews = N | N = 22  Age: 32-64 years  Gender: 11 males, 11 females  Nationality/ethnicity: Swedish | Disease duration: 5-42 years  Cohabitation: 18 cohabiting, 4 living alone  Employment status: 21 employed (13 full time, 4 part time, 4 sick leave), 1 unemployed |
| Kawi J (2012) | Chronic low back pain | Methodology:   - Surveys with open-ended questions | N = 110  Age: 19-86 years (Mean = 47)  Gender: 45 males, 65 females  Nationality/ethnicity: USA | Disease duration: 0.25-50 years (Mean = 10.88)  Cohabitation: N/A  Employment status: N/A |
| Kimani K N et al. (2018) | Advanced heart failure | Methodology:   - Interviews with patients or their carers if the patient became too ill   Interview duration: 25-125 minutes  Repeated interviews = Y, 3-6-month intervals | N = 18  Age: N/A  Gender: 8 males, 10 females  Nationality/ethnicity: Kenyan | Disease duration: N/A  Cohabitation: N/A  Employment status: N/A |
| Kjeken I et al. (2013) | Hand osteoarthritis | Methodology:   - Semi-structured interviews   Interview duration: N/A  Repeated interviews = N | N = 125  Age: 18-80 years  Gender: 3 males, 122 females  Nationality/ethnicity: Norwegian | Disease duration: Mean = 14.1 years  Cohabitation: 42 living alone  Employment status: 50 employed |
| Kneck A et al. (2014) | Type 1 or 2 diabetes | Methodology:   - Interviews (either at the hospital, participant’s workplace, or participant’s home)   Interview duration: 45-70 minutes  Repeated interviews = Y, 1-2 months, 1 year, and 3 years after diagnosis | N = 13  Age: 26-65 years (Mean = 44)  Gender: 9 males, 4 females  Nationality/ethnicity: Swedish | Disease duration: N/A  Cohabitation: 10 cohabiting, 3 living alone  Employment status: 11 employed, 2 unemployed |
| Levin-Zamir D et al. (2016) | Type 2 diabetes | Methodology:   - Focus groups with patients   Focus group duration: N/A | N = 56  Age: ≥20 years  Gender: 29 males, 27 females  Nationality/ethnicity: Israeli / of Muslim, non-Bedouin Arab heritage | Disease duration: N/A  Cohabitation: N/A  Employment status: N/A |
| Liden E et al. (2015) | Medically unexplained symptoms | Methodology:   - Narrative interviews   Interview duration: 21-80 minutes  Repeated interviews = N | N = 10  Age: 24-61 years  Gender: 3 males, 7 females  Nationality/ethnicity: Swedish / 3 Swedish, 7 migrant background | Disease duration: N/A  Cohabitation: N/A  Employment status: N/A |
| Lindgren I et al. (2018) | Persistent shoulder pain after stroke | Methodology:   - Semi-structured interviews at the stroke rehabilitation centre   Interview duration: 30-50 minutes  Repeated interviews = N | N = 13  Age: 57-77 years  Gender: 7 males, 6 females  Nationality/ethnicity: Swedish | Disease duration: 0.4-4.5 years (Mean = 2)  Cohabitation: 7 cohabiting, 6 living alone  Employment status: N/A |
| Lindquist H et al. (2015) | Cancer survivors with lymphedema | Methodology:   - Open interviews   Interview duration: 90-120 minutes  Repeated interviews = N | N = 8  Age: 45-71 years  Gender: All female  Nationality/ethnicity: Swedish | Disease duration: During treatment – 12 years after treatment  Cohabitation: N/A  Employment status: 6 employed, 2 unemployed |
| Martin F et al. (2013) | HIV-positive living with anti-retroviral therapy | Methodology:   - Interviews   Interview duration: N/A  Repeated interviews = Y, every month for 8 months | N = 20  Age: 26-58 years  Gender: 10 males, 10 females  Nationality/ethnicity: Ugandan | Disease duration: N/A  Cohabitation: 9 married / 11 unmarried  Employment status: N/A |
| Matima R et al. (2018) | HIV-positive living with Type 2 diabetes | Methodology:   - Semi-structured interviews   Interview duration: N/A  Repeated interviews = N | N = 10  Age: ≥18 years  Gender: 5 males, 5 females  Nationality/ethnicity: South African | Duration of diseases: 8.2 years (HIV) / 9.4 years (Type 2 diabetes)  Cohabitation: 7 married, 3 un-married  Employment status: N/A |
| Matthie N et al. (2015) | Sickle cell disease | Methodology:   - Semi-structured interviews   Interview duration: 30 minutes  Repeated interviews = Y | N = 29  Age: Mean = 25.8 years  Gender: 6 males, 23 females  Nationality/ethnicity: USA | Disease duration: Lifelong  Cohabitation: 21 living alone  Employment status: 10 employed |
| McQuoid J et al. (2017) | Chronic kidney disease | Methodology:   - Diaries over two days - Interview at the participant’s home, workplace, university campuses, dialysis clinics, or cafes   Interview duration: 60-180 minutes  Repeated interviews = N | N = 26  Age: 18-85 years  Gender: 13 males, 13 females  Nationality/ethnicity: Australian / European (n=21), Burmese (n=1), Filipino (n=1), Indian (n=1), Laos (n=1), South African (n=1) | Disease duration: N/A  Cohabitation: 7 living alone  Employment status: 13 employed |
| Moore L et al. (2015) | Coronary heart disease | Methodology:   - Written diary for 2 weeks - 4 interviews at the participant’s home   Interview duration: N/A  Repeated interviews = N | N = 21  Age: 60-85 years  Gender: 12 males, 9 females  Nationality/ethnicity: UK | Disease duration: N/A  Cohabitation: 7 living alone  Employment status: 1 employed |
| Mousavizadeh S N et al. (2017) | Type 2 diabetes | Methodology:   - In-clinic interviews - In-home interviews   Interview duration: 40-120 minutes  Repeated interviews = N | N = 21  Age: 36-67 years  Gender: N/A  Nationality/ethnicity: Iranian | Disease duration: 2-16 years  Cohabitation: N/A  Employment status: N/A |
| O’Conor R et al. (2017) | Asthma | Methodology:   - Focus groups (6 groups with 31 total participants)   Focus group duration: 90 minutes | N = 31  Age: Mean = 67.7 years  Gender: 4 males, 27 females  Nationality/ethnicity: USA (New York City) / 68% Hispanic, 10% non-Hispanic black | Disease duration: 29.6 years  Cohabitation: N/A  Employment status: N/A |
| Pauling J D et al. (2018) | Systemic sclerosis | Methodology:   - Focus groups (max. 9 people each)   Focus group duration: 60 minutes | N = 40  Age: Mean = 56.6 years  Gender: 6 males, 34 females  Nationality/Ethnicity: USA and UK / 26 white, 12 black, 2 Hispanic | Disease duration: Mean = 10.5 years  Cohabitation: N/A  Employment status: N/A |
| Peoples H et al. (2017) | Advanced cancer | Methodology:   - Interviews at the participant’s home   Interview duration: N/A  Repeated interviews = N | N = 73  Age: Mean = 68.3 years  Gender: 39 males, 34 females  Nationality/ethnicity: Danish | Disease duration: N/A  Cohabitation: N/A  Employment status: 9 employed |
| Rintala T M et al. (2013) | Type 1 diabetes | Methodology:   - Interviews   Interview duration: 40–120 minutes  Repeated interviews = N | N = 19  Age: 28-65 years (Mean = 42)  Gender: 1 male, 18 females  Nationality/ethnicity: Finnish | Disease duration: 2-58 years (Mean = 28)  Cohabitation: 17 either married or cohabiting, 7 with no children, 5 with grown up (adult) children  Employment status: N/A |
| Roberts A R et al. (2017) | One or more medically diagnosed chronic conditions | Methodology:   - 2 Surveys (mailed) (One survey sent at t=0 and another 6 months later) - Quantitative questionnaires | N = 130  Age: 65-88 years  Gender: All female  Nationality/ethnicity: USA / 68.1% white, 29.7% black, 2.2% Hispanic | Disease duration: N/A  Cohabitation: 25% married/has a partner  Employment status: N/A |
| Robinson K et al. (2018) | Chronic obstructive pulmonary disease | Methodology:   - Semi-structured interviews   Interview duration: 7-67 minutes  Repeated interviews = N | N = 18  Age: N/A  Gender: 12 males, 6 females  Nationality/ethnicity: Australian (New South Wales) | Disease duration: N/A  Cohabitation: N/A  Employment status: N/A |
| Stridsman C et al. (2013) | Chronic obstructive pulmonary disease | Methodology:   - Semi-structured interviews   Interview duration: 20-50 minutes  Repeated interviews = N | N = 20  Age: Mean = 69.1 years  Gender: N/A  Nationality/ethnicity: Swedish | Disease duration: N/A  Cohabitation: N/A  Employment status: 2 employed |
| Swenne C L et al. (2017) | Peritoneal carcinomatosis | Methodology:   - Open interview (telephone)   Interview duration: 23-78 minutes  Repeated interviews = N | N = 16  Age: 39-70 years (Mean = 56)  Gender: 7 males, 9 females  Nationality/ethnicity: Swedish | Disease duration: N/A  Cohabitation: 14 cohabiting, 2 living alone  Employment status: 10 employed, 6 unemployed |
| Thompson M (2014) | Type 1 or 2 diabetes | Methodology:   - Semi-structured interviews (used participant-generated photographs to elicit and guide answers during interviews)   Interview duration: N/A  Repeated interviews = N | N = 8  Age: 36-78 years  Gender: 5 males, 3 females  Nationality/ethnicity: USA / 100% Caucasian | Disease duration: 0.5-35 years  Cohabitation: N/A  Employment status: N/A |
| Walthall H et al. (2016) | Chronic heart failure | Methodology:   - Semi-structured interview   Interview duration: 45-90 minutes  Repeated interviews = N | N = 25  Age: 53-86 years  Gender: 15 males, 10 females  Nationality/ethnicity: UK | Disease duration: N/A  Cohabitation: N/A  Employment status: N/A |
| Westra B L et al. (2013) | Chronic diseases | Methodology:   - Semi-structured interviews conducted in patient’s home   Interview duration: N/A  Repeated interviews = N | N = 30  Age: ≥65 years  Gender: 3 males, 27 females  Nationality/ethnicity: USA | Disease duration: N/A  Cohabitation: N/A  Employment status: N/A |
| Wilson O et al. (2017) | Rheumatoid arthritis (associated foot problems) | Methodology:   - Semi-structured interviews   Interview duration: 35-60 minutes  Repeated interviews = N | N = 12  Age: Mean = 56 years  Gender: 5 males, 7 females  Nationality/ethnicity: UK | Disease duration: 12 years  Cohabitation: N/A  Employment status: N/A |
| Zhang L et al. (2016) | Atrial fibrillation | Methodology:   - Semi-structured interviews (using the ‘think-aloud’ technique on patient responses to ‘Quality of Life’ instruments)   Interview duration: N/A  Repeated interviews = N | N = 12  Age: N/A  Gender: 8 males, 4 females  Nationality/ethnicity: Australian | Disease duration: N/A  Cohabitation: N/A  Employment status: 6 employed |
