# Supplementary material for: Patient Work and Their Contexts: Scoping Review
Source: J Med Internet Res. 2020 Jun 2;22(6):e16656. doi: 10.2196/16656 (PMC7298639; doi:10.2196/16656)
Supplement: Multimedia Appendix 4 [file jmir_v22i6e16656_app4.docx]

Out of the 9 review articles included, 6 were systematic reviews, 2 were narrative reviews, and 1 article was described by the authors as a qualitative meta-synthesis review.

Similar to trends observed in primary research articles, the reviews were predominantly produced by countries with a northern European culture, with the US producing 3 reviews, the UK producing 2, followed by Australia (2), Denmark (1), and Norway (1). Also similar to that observed in primary research articles, 4 out of the 9 reviews focussed on patient work surrounding cardiovascular diseases and related symptoms. The rest of the reviews focused on chronic disease (2), pain (1), respiratory diseases (1), and neurological diseases (1).

| **Study author(s) / year published** | **Health condition(s)** | **Methodology / duration** | **Sample size / characteristics** | |
| --- | --- | --- | --- | --- |
| Boehmer K R et al. (2016) | Chronic disease – multiple conditions | Systematic review  Databases = MEDLINE, EMBASE, PsycInfo, CINAHL  from Jan 2000 – May 2014 | N of studies = 110  N of participants = N/A  Nationality of studies: 48 (USA), 19 (UK) and a minority from elsewhere. | Study methods of papers:  focus groups, semi-structured interviews, open-ended interviews. |
| Bratzke L C et al. (2015) | Adults with multimorbidity | Narrative review - Databases searched include PubMed, PyschINFO, CINAHL Plus, and SocINDEX (Inception – December 2013) | N of studies: 13  N of participants: N/A  Nationality of studies: 8 (USA)5 studies in Europe, | Study methods of papers:  Articles included focus groups, semi-structured interviews and surveys |
| Hunt T et al. (2014) | Chronic obstructive pulmonary disease (COPD) | Systematic review of peer reviewed articles, reporting at least one free-living activity engaged in by people with COPD (activity type, duration and/or frequency)  Time frame: Initial search in April 2012, updated search conducted in June 2013  Databases searched: Medline, Embase, EBSCO Host, Science Direct, SAGE Journals Online, and Scopus | No. of studies = 26  No. of participants: 10 – 5,314 participants per study  Nationality/ethnicity: UK (n=3), Turkey (n=2), Sweden (n=1), Belgium (n=3 studies), Denmark (n=1), Brazil (n=7), USA (n=2 studies), Austria (n=1), Switzerland (n=2), Venezuela (n=1), Ireland (n=1), Spain (n=1). (Australian paper) | Study methods of papers:  Face-to-face interview (n=5 studies), questionnaire (n=6 studies), activity monitoring (n=15 studies), self-reported diary (n=1 study), telephone interview (n=1 study) |
| Learmonth Y C et al. (2016) | Multiple sclerosis | Systematic review of literature to the consequences or determinants of physical activity in multiple sclerosis  Databases with timeframes: Cinahl, Embase (1988-Feb 2014), Medline (1950-Feb 2014), PsycINFO (1967-Feb 2014), PubMed (1950-Feb 2014), and Web of Science (1900-Feb 2014) | No. of studies = 19  No. of participants: 6 – 30 per study  Nationality of studies: N/A (US paper) | Study methods used in papers:  Focus groups (n=4 studies), face-to-face interview (n=14 studies), telephone interview (n=2 studies) |
| Schjoedt I et al. (2016) | Heart failure | Systematic review of qualitative studies regarding the experiences and management of fatigue in adults with heart failure  Databases: Medline (PubMed), Embase, SveMedþ, Cochrane Library, CINAHL, PsycINFO, Web of Science, Bibliotek.dk, ProQuest Dissertations and Theses Database, MEDNAR, Google.com, Bibliotek.dk  Time frame: Searched in August 2012, updated in December 2014. Covering articles from January 1995 to December 2014 | Total = 5 studies  No. of participants: 2 – 26 participant per study  Nationality of studies: Sweden (n=2), USA (n=2), Denmark (n=1) (Danish paper) | Study methods:  100% face-to-face interviews |
| Coventry P A et al. (2015) | Hypertension (4 studies), high blood pressure (3 studies), depression (3 studies), diabetes (2 studies), arthritis (1 study) | Systematic review -Databases: MEDLINE, Embase, PsycINFO, CINAHL, and ASSIA from inception to April 2015 | No. of studies: 19 studies  No. of participants: 7-100  Nationality of studies: U.S. = 10, UK = 6, Canada = 1, Amsterdam = 1, Germany = 6 (UK paper)  Age range across all studies: 30-97 (10 studies recruited participants between age range 40-80+) | Study methods of papers:  Semi-structured interview (n=13 studies), focus groups (n=5), combination of both (n=1). |
| Jani B et al. (2013) | Chronic heart failure | Limitations: English language, year of publication 2000 onwards  Systematic review of databases: Scopus, CINAHL, Embase, Medline, PsycINFO and citation searching | No. of studies: 16 articles  No. of participants: N/A  Nationality of studies: N/A | Study methods of papers:  N/A  More details of review protocol have been published elsewhere references [13, 14] in the paper |
| Johnston C M (2015) | Complex Regional Pain Syndrome | Narrative review of databases: ProQuest, EBSCO, Informit, Scopus/Science Direct and Web of Science, Medline, CINHAL, Google Scholar  Limitations: Articles from 1998 – 2015, English language | No. of studies: 12 articles  No. of participants: N/A  Nationality of studies: N/A (Australian paper) | Study methods of papers:  N/A |
| Eilertsen G et al. (2012) | Poststroke fatigue | Databases: PubMed, CINAHL, PsycINFO, Embase  Timeframe: Inception to April 2012 | No. of studies: 12 papers  No. of participants: 229 participants  Nationality of studies: USA and Europe (Norwegian paper) | Study methods of papers:  Semi structured interviews, mixed qualitative and quantitative interviews, focus group interviews, grounded theory interviews, descriptive interviews, phenomenography interviews |
