# Supplementary material for: Patient Work and Their Contexts: Scoping Review
Source: J Med Internet Res. 2020 Jun 2;22(6):e16656. doi: 10.2196/16656 (PMC7298639; doi:10.2196/16656)
Supplement: Multimedia Appendix 5 [file jmir_v22i6e16656_app5.docx]

| **Study**  **author(s) /**  **year published** | | **Work activity** | |  | **Contextual factor(s)** |  | | |  |
| --- | --- | --- | --- | --- | --- | --- | --- | --- | --- |
| Apps L D et al. (2015) | | - Monitor signs & symptoms (peak flow and pacing) | - Taking treatment | | - Nature of disease (debilitating breathlessness, unpredictability of symptoms) - Emotional state (fear of inducing symptoms) | - Heath education (Understanding of disease; perceptions of asthma by others) - Attitudes & beliefs | | |  |
| Arman M (2015) | | - Planning (Rest breaks) | - Seek medical help - Creating mental coping strategies | | - Social values & expectations - Emotional state - Quality of interdisciplinary care | - Attitudes & beliefs - Quality of care (health professionals) | | |  |
| Au T S et al. (2014) | | - Pro-active management of risks (e.g. reading less) - Deliberate distraction | - Seek medical help - Consult complementary therapy (Chinese herbal oil, self-massage) - Use & maintain assistive devices (cold or warm compression, using medical pads) | | - Nature of disease - Quality of care (health professionals) - Health education (about the condition) - Quality of care (government) – limited access to specialist care | - Emotional state (fear of medication effects) - Finance - Social support | | |  |
| Blandford A et al. (2015) | | - Planning (for self-dialysis) - Use & maintain assistive devices | - Learn about the disease (educating oneself about and adapting machines to better suit their individual needs) | | - Health education (condition, use of home haemodialysis) | - Quality of care (government) – having reliable home care team | | |  |
| Boehmer K R et al. (2016) | | - Planning (normalising self-care into lifestyle/routine) - Creating mental coping strategies | - Medication management e.g. taking less if necessary - Hire professional help (meals, transport) | | - Emotional state - Social support (positive and negative) - Quality of care (health professionals) - Finance - Access to public support facilities (transport) | - Health education - Social values & expectations - Quality of care (government) - Attitudes and beliefs (very independent personalities disliked being told what to do) | | |  |
| Bowling C B et al. (2017) | | - Planning (writing questions to ask doctor) - Monitor signs & symptoms (measuring daily water intake) | - Medication management (using pillboxes) - Seek medical help - Self-manage co-morbidities | | - Nature of condition - Quality of care (health professionals) - Attitudes and beliefs (personality) | - Social values & expectations - Social support - Quality of interdisciplinary care | | |  |
| Bratzke L C et al. (2015) | | - Planning (normalising self-care into lifestyle/routine) | - Medication management - Seek medical help | | - Nature of condition - Emotional state (fear of medication effects) - Quality of care (health professionals) – rapport with health professionals - Finance - Social support | - Attitudes & beliefs - Social values & expectations (avoiding use of cane as ‘the cane means I am ill’) - Complexity of treatment - Health education - Access to public support facilities | | |  |
| Browne C et al. (2015) | | - Creating mental coping strategies - Pro-active management of risks (frequently going toilet to avoid leaking) | - Planning - Monitor signs & symptoms - Conduct exercise (pelvic floor exercises, stimulating the bladder control sphincter) | | - Nature of condition - Emotional state (fear of being burden to others) - Health education - Social values & expectations | - Quality of care (government) - External environment - Attitudes & beliefs - Social support | | |  |
| Bukhave E B et al. (2013) | | - Creating mental coping strategies (not peeling potatoes when cooking due to arthritic hands - Planning | - Pro-active management of risks (quit an activity) - Ask for help from family & friends - Alter the physical environment (modified lawn mower) | | - Access to public support facilities (transport) - Nature of condition - Social support - Change in ability to work | - Social values/expectations - Finance - External environment - Health education | | |  |
| Burnett K et al. (2018) | | - Learn about the disease (about the condition) - Medication management (pausing or changing treatment) - Use & maintain assistive devices (oxygen tank) - Diet control - Search for & attend patient support groups (meeting others with IPF in seminars, peer support groups, online forums etc.) | - Conduct exercise - Pro-active management of risks (vaccinations, increased sun protection) - Self-manage co-morbidities (taking anti-diarrhoeal, cough lozenges) - DIY symptom management tools (sucking ice cubes, handheld fans) - Consult complementary therapy (Echinacea, salt pipes) | | - Health education (condition) - Quality of care (health professionals) – health professionals’ knowledge - Finance - Quality of interdisciplinary care | - Changes in ability to work - Attitudes and beliefs (believe a more positive health professional is better) - Emotional state (fear of medication side effects) | | |  |
| Cameron M H et al. (2013) | | - Pro-active management of risks (turn on the lights when moving around the home - Ask for help from family & friends | - Learn about the disease (at least once a year, ask health care professional about ways to reduce fall risk) | | - Emotional state (fear of falling) | - Access to public support facilities | | |  |
| Chen K et al. (2016) | | - Pro-active management of risks - Monitor signs & symptoms (diet, exercise, heart rate) | - Self-manage co-morbidities - Seek medical help - Conduct exercise | | - Emotional state - Social support - External environment | - Health education - Quality of care (government) | | |  |
| Cheung M M Y et al. (2018) | | - Pro-active management risks (avoid triggers of asthma exacerbations e.g. crabs, frozen food, flowers) - Creating mental coping strategies | - Adapt to social values & expectations (not sharing personal health details, not using asthma medication in public) | | - Nature of disease - Attitudes and beliefs (wants to be able to participate in recreational interests e.g. singing, hikes) | - Social support network (of family and friends) - Health education (about condition) | | |  |
| Close C et al. (2015) | | - Ask for help from family & friends - Seek medical help - Medication management (avoiding particular medications during pregnancy) | - Creating mental coping strategies - Planning (normalising self-care into lifestyle/routine) - Pro-active management of risks (taking a bath regularly in response to back pain) - Conduct exercise (swimming) | | - Health education - Emotional state (fear of medication use) | - Quality of care (health professionals) - Attitudes & beliefs | | |  |
| Cobussen-Boekhorst H et al. (2016) | | - DIY symptom management tools (using a mirror for catheterisation) - Taking treatment (washing hands, preparing the catheter, washing the genitals, where to leave the catheter afterwards) | - Planning (social life around catheterisation schedule) - Pro-active management of risks (Catheterise one extra per day to avoid the need to find outdoor toilets) - Ask for help from family & friends (with catheterisation) | | - Quality of care (health professionals) – access to professionals’ advice via phone call - Health education (workplace colleagues) - Access to public support facilities (small toilets, poor lighting, sink being outside of toilet cubicle) | - Quality of care (government) – intermittent catheterisation preferred over indwelling catheters - Attitudes and beliefs | | |  |
| Coventry P A et al. (2015) | | - Planning (strict vs flexible routine) - Learn about the disease (listening to one’s body) | - Creating mental coping strategies (going outdoors) - Consult complementary therapy - Medication management (changing doses of medicine) - Taking treatment (Pain management via over-the-counter medicine) | | - Complexity of treatment - Social support - Attitudes and beliefs (want to control symptoms and to remain independent) | - Emotional state (fear of medication side effects) - Social values & expectations | | |  |
| Czuber-Dochan W et al. (2013) | | - Learn about the disease (specific triggers that lead to fatigue worsening) - Planning (a day off, normalising self-care, ‘cat naps’) | - Deliberate distraction - Creating mental coping strategies (making errors at work) - Diet control | | - Nature of disease - Social support (family) - Attitudes & beliefs | - Health education (workplace) - Change in ability to work - Quality of care (health professional) | | |  |
| Dahlviken R M et al. (2015) | | - Taking treatment (sedative/analgesic drugs, relaxing) - Conduct exercise - Diet control - Pro-active management of risks | - Creating mental coping strategies (normalising selfcare) - Planning (sick leave, working part-time, reducing workload) - Seek medical help (frequently) | | - Attitudes and beliefs (believe the health condition can get better) - Quality of care (health professionals) | - Nature of disease - Health education (self and others about condition) | | |  |
| dal Bello-Haas et al. (2014) | | - Conduct exercise - Creating mental coping strategies - Self-manage co-morbidities | - Diet control - Deliberate distraction - Seek medical help | | - External environment | - Quality of care (government) | | |  |
| Dehghanzadeh S et al. (2017) | | - Seek medical help (CRT replacement) - Planning (appointments and transportation) - Ask for help from family & friends | - Pro-active management of risks - Creating mental coping strategies - Use & maintain assistive devices | | - Emotional state (anxiety towards treatment e.g. device malfunction) - Finance - Quality of care (government) - Change in ability to work | - Cultural influences - Health education (condition, device) - Social support - Quality of care (health professionals) | | |  |
| Dial M et al. (2018) | | - DIY symptom management tools (using a commode as a walker at home) - Use & maintain devices (hand-held shower head) | - Planning - Alter the physical environment - Pro-active management of risks (skin infections) | | - Social support - Quality of care (health professionals) – rapport with health professionals - Nature of disease - Access to public support facilities (e.g. handrails) | - Health education - External environment | | |  |
| Eilertsen G et al. (2012) | | - Creating mental coping strategies (Irritability, impaired stress tolerance) | - Pro-active management of risks (increased sensitivity to sound and light, taking naps) | | - Nature of disease - Change in energy levels | - Health education (condition, family, workplace) | | |  |
| Ferreira S L et al. (2013) | | - Planning (to have treatment on time) - Taking treatment (blood transfusions, self-care for wounds, prostheses) | - Ask for help from family & friends - Seek medical help (mainly due to pain) - Alter the physical environment (temperature control) | | - Health education (health professionals on the disease) - Social values & expectations - Attitudes and beliefs - Quality of care (government) | - Quality of care (health professionals) - Change in ability to work | | |  |
| Flurey C A et al. (2013) | | - Planning (daily tasks, naps) - Creating mental coping strategies (adjusting to the new normal) | - Seek medical help e.g. RA flares - Ask for help from family & friends - Taking treatment - DIY symptom management tools (icing painful area) | | - Social support (family, friends, medical team, other RA patients) - Nature of disease - Emotional state (e.g. social withdrawal) |  | | |  |
| Franklin Z C et al. (2016) | | - Creating mental coping strategies (mental coping strategies) - Ask for help from family & friends | - Planning - Teach others about their health - Medication management (decreasing doses) - Seek medical help | | - Social support - Nature of condition - Health education (self and others) - Emotional state (fear of medication use) | - Attitudes and beliefs (sceptical) - Access to public support facilities - Quality of care (health professionals) | | |  |
| Fritz H (2014) | | - Planning (Normalising selfcare) - Creating mental coping strategies | - Taking treatment - Monitor signs & symptoms (blood glucose) - Diet control | | - Social values & expectations - Attitudes and beliefs (convenience of care) | - Finance - Access to public support facilities | | |  |
| Gallacher K I et al. (2018) | | - Learn about the disease (making sense of symptoms) - Planning - Seek medical help | - Hire professional help - Pro-active management of risks (gauging physical capacity) - Medication management - Diet control | | - Health education - Quality of care (health professionals) - Access to public support facilities (transport) - Social values & expectations - Quality of care (government) | - Social support - Complexity of treatment - Finance - Emotional state (fear of side effects of medication) | | |  |
| Gerrish K et al. (2013) | | - Creating mental coping strategies |  | | - Quality of care (health professional) - Health education (health professionals, community) | - Social values & expectations - Change in ability to work - Language barriers - Social support | | |  |
| Graffigna G et al. (2013) | | - Creating mental coping strategies (normalising self-care, lack of compliance) | - Conduct exercise - Monitor signs & symptoms (blood glucose and blood pressure) - Diet control | | - Health education (condition) | - Quality of care (health professionals) – rapport at the professional level | | |  |
| Graham L J et al. (2013) | | - Ask for help from family & friends - Conduct exercise - Pro-active management of risks (avoiding the sun, wearing sunscreen, personal hygiene) | - Taking treatment (as prescribed and finding and using cold and flu medication) - Diet control | | - Access of public support facilities (transport) - Social support (exercise buddies) - Social values & expectations (eating as a social activity) | - Attitudes and beliefs (Perception of exercise as very broad and unstructured activity, activity must be “something that you can enjoy”, want to remain independent) | | |  |
| Halls S et al. (2014) | | - Conduct exercise (managing stiffness by moving, stretching, moving while still in bed, supporting or manipulating joints) - DIY symptom management tools (use of hot showers, ice packs to relieve stiffness) | - Planning (e.g. scheduling MRI scans) - Adapt to social values & expectations (avoiding particular clothing) | | - Attitudes and beliefs (cause of illness and its impact) | - External environment (wet and cold) | | |  |
| Hammarlund C S et al. (2017) | | - Pro-active management of risks - Learn about the disease - Hire professional help - Planning - Use & maintain assistive devices | - Conduct exercise - Creating mental coping strategies - Deliberate distraction | | - Change in energy levels - Nature of diseases - Health education | - External environment (weather and climate conditions) - Access to public support facilities - Social values & expectations (stigma) | | |  |
| Holden R J et al. (2015) | | - Seek medical help - Creating mental coping strategies (making sense of new situations) | - Monitor signs & symptoms (weight) - Diet control (sodium-restricted diet) - Self-manage co-morbidities | | - Nature of disease - Emotional state (fear of medication side effects) - Finance - Complexity of treatment - External environment | - Health education (condition, self-care) - Attitudes & beliefs - Social support - Quality of care (government) | | |  |
| Holden R J et al. (2017) | | - Taking treatment - Monitor signs & symptoms - Teach others about their health | - Planning (following healthcare routines, daily plans, managing life demands) - Seek medical help | | - External environment (home, distance to areas of interest) - Emotional state (fear of medication side effects and being a burden) - Access to public support facilities (mobile app) | - Social support (in/out of home) - Social values & expectations - Cultural influences - Finance - Quality of care (government) | | |  |
| Hunt T et al. (2014) | | - Diet control | - Planning (travelling to see health professionals / use health services) | | - Nature of the disease |  | | |  |
| Jacobsson L R et al. (2017) | | - Learn about the disease - Diet control - Consult complementary therapy - Planning - Pro-active management of risks | - Taking treatment - Deliberate distraction | | - Emotional burden | - Social values & expectations - Change in energy levels | | |  |
| Jani B et al. (2013) | | - Diet control (reducing salt and fluid intake) - Monitor signs & symptoms (weight) | - Pro-active management of risks (planning outings around frequent urination schedule - Seek medical help | | - Health education (health professionals, family and friends) | - Quality of interdisciplinary care | | |  |
| Johnston C M (2015) | | - Deliberate distraction (to deal with constant pain: TV, alcohol, cigarettes, mindfulness, meditation, praying, pacing) - Planning (activities) | - Seek medical help (attending rehab programme or patient coaching interventions) - Learn about the disease - Creating mental coping strategies | | - Nature of disease | - Health education (condition) - Attitudes and beliefs (want to legitimise their condition to others) | | |  |
| Kawi J (2012) | | - Taking treatment - Conduct exercise - Planning (travelling to see health professionals / use health services) - Consult complementary therapy | - Diet control - Pro-active management of risks - DIY symptom management tools (applying own heat and cold applications when necessary) | | - Quality of interdisciplinary care | - Quality of care (health professionals) – rapport with health professional | | |  |
| Kimani K N et al. (2018) | | - Creating mental coping strategies |  | | - Nature of condition - Social support (support networks) - Health education (condition, selfcare strategies) | - Finance | | |  |
| Kjeken I et al. (2012) | | - DIY symptom management tools (most frequently used for opening packing and cutting food) - Pro-active management of risks (avoid certain activities) | - Diet control - Ask for help from family & friends - Creating mental coping strategies - Conduct exercise | | - Emotional state - Quality of care (health professional) | - Health education | | |  |
| Kneck A et al. (2014) | | - Learn about the disease - Planning | - Deliberate distraction | | - Emotional state - Health education | - Finance | | |  |
| Learmonth Y C et al. (2015) | | - Conduct exercise |  | | - Access to public support facilities - External environment - Quality of interdisciplinary care - Quality of care (health professionals) – rapport with health professional - Finance - Social values & expectations | - Emotional state - Change in energy levels - Nature of diseases - Health education | | |  |
| Levin-Zamir D et al. (2016) | | - Planning (travelling to see health professionals / use health services) |  | | - Nature of diseases - Health education - Social values & expectations | - Finance - Social support - Access to public support facilities - Cultural influences | | |  |
| Liden E et al. (2015) | | - Planning | - Deliberate distraction | | - Nature of diseases - Change in energy levels | - Language barrier (for immigrants) - Emotional state | | |  |
| Lindgren I et al. (2018) | | - Taking treatment | - Planning (travelling to see health professionals / use health services) | | - Nature of diseases | - Emotional state - Health education | | |  |
| Lindquist H et al. (2015) | | - Conduct exercise - Planning | - Use & maintain assistive devices | | - Social support - Change in energy levels | - Health education | | |  |
| Martin F et al. (2013) | | - Taking treatment - Planning - Monitor signs & symptoms - Pro-active management of risks - Medication management (side effects) | - Teach others about their health - Ask for help from family & friends - Adapt to social values & expectations - Search for & attend patient support groups - Diet control - Learn about the disease | | - Quantity of care (government) – resources & medical supplies - Quality of care (health professionals) - Attitudes and beliefs - Health education - Finance | - Social support - Emotional state - Social values & expectations | | |  |
| Matima R et al. (2018) | | - Self-manage co-morbidities - Planning - Diet control | - Taking treatment - Conduct exercise - Adapt to social values & expectations | | - Quantity of care (government) – resources & medical supplies - Quality of care (health professionals) - Social support | - Health education - Finance | | |  |
| Matthie N et al. (2015) | | - Pro-active management of risks - Taking treatment - Creating mental coping strategies (accepting their life, religious support) | - Learn about the disease - Planning - Diet control | | - Social support - Finance - Health education - Emotional state | - Quality of care (health professionals) – rapport with health professional - Social values & expectations | | |  |
| McQuoid J et al. (2017) | | - Planning - Conduct exercise | - Diet control - Taking treatment | | - Social support - Quality of interdisciplinary care | - Quantity of care (government) – Structure of the healthcare system | | |  |
| Moore L et al. (2015) | | - Search for & attend patient support groups - Ask for help from family & friends - Use & maintain assistive devices - Taking treatment | - Monitor signs & symptoms - Conduct exercise - Diet control - Learn about the disease - Pro-active management of risks | | - Nature of disease - Social support - Health education - Attitudes and beliefs | - Quality of care (health professionals) – rapport with health professional - Finance - Emotional state - Quality of interdisciplinary care | | |  |
| Mousavizadeh S N et al. (2017) | | - Taking treatment - Diet control - Planning (health professional meetings / use health services) | - Pro-active management of risks - Monitor signs & symptoms | | - Health education - Nature of diseases - Social values & expectations | - Finance - Social support - Attitudes and beliefs - Cultural influences | | |  |
| O’Conor R et al. (2017) | | - Planning - Pro-active management of risks - Taking treatment - Ask for help from family & friends | - Monitor signs & symptoms - Use & maintain assistive devices - Hire professional help | | - Nature of disease - External environment - Quantity of care (government) – Structure of the healthcare system | - Health education - Emotional state - Social values & expectations - Attitudes and beliefs - Social support | | |  |
| Pauling J D et al. (2018) | | - Adapt to social values & expectations (wear extra clothing/gloves in ambient conditions due to cutaneous discoloration) | - Proactive management of risks (keeping environment warm or avoiding cold exposure) | | - Emotional state - External environment (Cold exposure) - Changes in ability to work | - Health education (colleagues and family members) - Nature of disease | | |  |
| Peoples H et al. (2017) | - Planning - Hire professional help - Ask for help from family & friends | | - Use & maintain assistive devices | | - Nature of diseases - Emotional state | | - Finance - Social support - Change in energy levels |  |  |
| Rintala T M et al. (2013) | | - Pro-active management of risks (prevention of hypoglycaemia) - Monitor signs & symptoms (Blood glucose, times of insulin injections) | - Diet control - Teach others about their health (how they can take care of him/her) - Medication management (Adjusting insulin dose) | | - Social support (depend on family members as well as responsibility to look after children) | - Attitudes and beliefs (places great importance on independence) | | |  |
| Roberts A R et al. (2017) | - Medication management (side effect) - Taking treatment - Use & maintain assistive devices | | - Monitor signs & symptoms - Learn about the disease - Conduct exercise - Diet control - Planning | | - Social support - Nature of diseases - Finance | | - Change in energy levels - Attitudes and beliefs | | |
| Robinson K et al. (2018) | | - Creating mental coping strategies (normalising self-care) - Seek medical help (ED when necessary) | - Use & maintain assistive devices (oxygen therapy) - Teach others about their health (action plan) | | - Quality of care (government) – quick access to GP or community providers - External environment | - Quality of care (health professionals) | | |  |
| Schjoedt I et al. (2016) | - Pro-active management of risks - Planning | | - Use & maintain assistive devices | | - Change in energy levels - Nature of diseases | | - Emotional state | | |
| Stridsman C et al. (2013) | - Planning - Conduct exercise - Ask for help from family & friends - Taking treatment | | - Pro-active management of risks - Learn about the disease - Search for & attend patient support groups | | - External environment - Nature of diseases - Social support | | - Health education - Quality of care (health professionals) – rapport with health professional - Emotional state | | |
| Swenne C L et al. (2017) | - Search for & attend patient support groups | |  | | - Social values & expectations - Change in energy levels - Nature of diseases - Emotional state | | - Finance - Social support - Health education - Quality of interdisciplinary care | | |
| Thompson M (2014) | - Conduct exercise - Diet control - Monitor signs & symptoms - Pro-active management of risks - Planning | | - Learn about the disease - Use & maintain assistive devices - Taking treatment | | - Cultural influences - Social support | | - Social values & expectations - Health education | | |
| Walthall H et al. (2016) | - Planning - Pro-active management of risks - Use & maintain assistive devices | | - Taking treatment - Learn about the disease - DIY symptom management tools | | - Change in energy levels - Social values & expectations | | - Health education - Attitudes and beliefs | | |
| Westra B L et al. (2013) | | - DIY symptom management tools (having sponge baths) - Ask for help from family & friends - Use & maintain assistive devices (grab bars, chairs etc.) - Diet control | - Hire professional help (food delivery, laundry, cleaning) - Monitor signs & symptoms (blood glucose) - Medication management (pillboxes) | | - Attitudes and beliefs (convenience of self-care, self-regulation) - Complexity of treatment | - Social support (Support network) - Access of public support facilities (transport) - External environment | | |  |
| Wilson O et al. (2017) | | - Self-manage co-morbidities (applying heat and cold modalities, filing calluses) | - Seek medical help - Use & maintain assistive devices (insoles in shoes) | | - Nature of disease - Emotional state | - Finance - Quality of care (health professional) - Health education | | |  |
| Zhang L et al. (2016) | | - Creating mental coping strategies - Taking treatment - Seek medical help (frequently) - Self-manage co-morbidities | - Pro-active management of risks e.g. avoidance of activities that might cause bleeding - Diet control - Medication management (deal with side effects) | | - Emotional state (anxiety towards symptoms) - Health education (condition, medication) | - Attitudes and beliefs (believe in independence) - Social support - Quality of care (health professional) | | |  |
