# Supplementary material for: Patient Work and Their Contexts: Scoping Review
Source: J Med Internet Res. 2020 Jun 2;22(6):e16656. doi: 10.2196/16656 (PMC7298639; doi:10.2196/16656)
Supplement: Multimedia Appendix 6 [file jmir_v22i6e16656_app6.docx]

| **Patient work task** | **Description** |
| --- | --- |
| Planning | Organisational work that integrates health-related tasks into existing daily routine, and prospectively plans for patient work tasks in the future.  E.g. Scheduling doctor’s appointment mentally and on a calendar |
| Pro-active management of risks | Organisational work that consciously identifies health-related risks and takes action to mitigate such risks.  E.g. Avoids physical exercise due to arthritic pain |
| Deliberate distraction | Cognitive work that involves psychological escapism and avoids thinking about one’s health.  E.g. Choosing to not think about one’s own health in patients with chronic pain |
| Adapt to social values & expectations | Cognitive work that recognises social stigma towards one’s health condition or health-related tasks and takes action to fit one’s action or appearance to social norms.  E.g. Consciously avoids using asthma inhalers in public |
| Creating mental coping strategies | Cognitive work that adjusts one’s mindset to accept and cope with changes in health states.  E.g. Going through the 5 stages of grief after diagnosis and accepting the disease as a part of one’s new ‘normal’ |
| Learn about the disease | Cognitive and physical work that involves looking for information to educate oneself about the disease and trying to make sense of the information.  E.g. Asking healthcare professionals about what the disease means and trying to understand this information |
| Diet control | Physical work involving the purchase, preparation, and consumption of food.  E.g. Shopping for fresh fruit and vegetables |
| Taking treatment | Physical work involving taking medical treatment (including, but not limited to, oral medications, injections, eyedrops, undertaking surgeries, etc).  E.g. Ingesting over-the-counter medication |
| Conduct exercise | Physical work involving physically conducting exercise.  E.g. Swimming |
| Monitor signs & symptoms | Organisational work involving measuring and recording health signs and symptoms.  E.g. Measuring and recording blood pressure daily |
| Medication management | Organisational work that ensures the right dose of medication is taken at the right time  E.g. Using pillboxes to sort out doses for the entire week |
| Use & maintain assistive devices | Physical work involving purchasing, using, and maintaining health assistive devices such as wheelchairs, walking sticks, insulin injection pens, etc.  E.g. Buying and using shoe insoles |
| DIY symptom management tools | Physical work involving DIY (do-it-yourself) manufacturing of health assistive devices and subsequent use of them  E.g. Using store-bought ice packs to temporarily keep insulin cool |
| Self-manage co-morbidities | Physical work involving managing new symptoms without consulting medication professionals, involving self-diagnosis and purchasing medications and devices without professional input.  E.g. Assuming a new prescription medicine is giving one diarrhoea, then purchases and takes anti-diarrhoeal medication to self-manage it |
| Alter the physical environment | Physical work involving physically changing the broader physical environment around the self.  E.g. Change the temperature of the environment to help with arthritis management |
| Seek medical help | Collaborative physical work that seeks medical help from health professionals  E.g. Hospitalisation and seeing medical doctors |
| Ask for help from family & friends | Collaborative physical work that seeks medical help from family, friends, and acquaintances.  E.g. Asking for family help with catheterisation at home |
| Hire professional help | Collaborative physical work that seeks help from people who are neither health professionals nor family and friends. This includes paid professionals and volunteers.  E.g. Using a volunteer-run meal delivery service |
| Consult complementary therapy | Collaborative physical work that seeks help from complementary therapy practitioners  E.g. Attending traditional Chinese medicine clinics |
| Search for & attend patient support groups | Collaborative physical work that seeks for information or companionship from patient support groups or patient organisations  E.g. Attend peer support groups |
| Teach others about their health | Collaborative physical work that seeks to educate other people about one’s health  E.g. A diabetic patient teaching his children about what they should do in the case of him having a hypoglycaemic episode |
