# Supplementary material for: Patient Work and Their Contexts: Scoping Review
Source: J Med Internet Res. 2020 Jun 2;22(6):e16656. doi: 10.2196/16656 (PMC7298639; doi:10.2196/16656)
Supplement: Multimedia Appendix 7 [file jmir_v22i6e16656_app7.docx]

| **Contextual Factor** | **Definition** | **Example** |
| --- | --- | --- |
| **Micro-level contextual factors** | | |
| Nature of disease | How the specific symptoms of a condition affected patient work | Mobility limitation from osteoarthritis.  Unpredictable bladder dysfunction limited exercise. |
| Emotional state | Patients’ feelings that affect how they conduct their health-related behaviour | Negative emotions (such as fear towards side effects) led to patients avoiding medication, while positive feelings of accomplishment motivated patient to exercise more |
| Attitudes and beliefs | How perceptions and beliefs about the self and the external world affect health-related work | Patients who believed they can ‘win’ over the disease conducted more patient work, while symptoms that were perceived to be more severe received prioritisation |
| Change in energy level | The influence of fatigue | Fatigue wore down self-esteem, internal identity, and intellectual functions |
| Changes in the ability to work | How patients must change the way they act at work | Lost confidence and feeling guilty over their reduced capacity to work |
| Complexity of treatment | Combined level of complexity from one’s treatment | Patient is prescribed a multitude of tablets, some to be taken in the morning and some at night, and the patient finds it difficult to keep track of when to take what medication |
| **Meso-level contextual factors** | | |
| Health education | Level of health literacy the patient possesses and whether they have knowledgeable family and friends | Patient’s and their family members’ knowledge regarding their disease or treatment |
| Social support | The presence (or lack of) social support | Not having anyone to talk to when depressed by the health condition |
| Social values & expectations | How the patient is expected to behave in their society | Older male patients were not involved in making food-related decisions and had no desires to be involved |
| Finance | How finances enabled or limited patient work tasks | Funding treatments |
| External environment | The influence of the physical environment that patient lives in | Temperature, air quality, distance to clinics, etc |
| Language barrier | The patient spoke a language different to that spoken by the healthcare system | Migrant populations struggle to understand local doctors |
| **Macro-level contextual factors** | | |
| Quality of care (health professionals) | How the quality of care a patient receives from individual health professionals influenced patient work tasks | Efficient, honest doctors that provided effective care boosted motivation for patient work, while doctors who were dismissive made patients unmotivated |
| Quality of care (government) | Policies from geopolitical entities affected how one carried out patient work | Quality of medical supplies available in the country was unsatisfactory |
| Access to public support facilities | Whether support facilities were available in the public space | Unavailability of public transport to the hospital |
| Quality of interdisciplinary care | Patients receive different (sometimes conflicting) advice from various health disciplines | Heart failure patients with kidney disorders receive contradictory advice regarding amount of water consumption |
| Cultural influences | How specific cultural practices interfered with health | Arab communities have social pressures on guests to eat refreshments, even if they are on a sodium- or glucose-restriction diet |
